# Canadian Association of Gastroenterology Clinical Practice Guideline for the Management of Irritable Bowel Syndrome (IBS)

**DOI:** 10.1093/jcag/gwy071

**Published:** 2019-01-17

**Authors:** Paul Moayyedi, Christopher N Andrews, Glenda MacQueen, Christina Korownyk, Megan Marsiglio, Lesley Graff, Brent Kvern, Adriana Lazarescu, Louis Liu, William G Paterson, Sacha Sidani, Stephen Vanner

**Affiliations:** 1 Division of Gastroenterology, McMaster University, Hamilton, Ontario, Canada; 2 Division of Gastroenterology, University of Calgary, Calgary, Alberta, Canada; 3 Department of Psychiatry, University of Calgary, Calgary, Alberta, Canada; 4 Department of Family Medicine, University of Alberta, Edmonton, Alberta, Canada; 5 Unaffliated; 6 Department of Clinical Health Psychology, University of Manitoba, Winnipeg, Manitoba, Canada; 7 Department of Family Medicine, University of Manitoba, Winnipeg, Manitoba, Canada; 8 Division of Gastroenterology, University of Alberta, Edmonton, Alberta, Canada; 9 Division of Gastroenterology, University of Toronto, Toronto, Ontario, Canada; 10 Division of Gastroenterology, Queen’s University, Kingston, Ontario, Canada

**Keywords:** Clinical practice guidelines, Constipation, Diarrhea, Irritable bowel syndrome

## Abstract

**Background & aims:**

Irritable bowel syndrome (IBS) is one of the most common gastrointestinal (GI) disorders, affecting about 10% of the general population globally. The aim of this consensus was to develop guidelines for the management of IBS.

**Methods:**

A systematic literature search identified studies on the management of IBS. The quality of evidence and strength of recommendations were rated according to the Grading of Recommendation Assessment, Development and Evaluation (GRADE) approach. Statements were developed through an iterative online platform and then finalized and voted on by a multidisciplinary group of clinicians and a patient.

**Results:**

Consensus was reached on 28 of 31 statements. Irritable bowel syndrome is diagnosed based on symptoms; serological testing is suggested to exclude celiac disease, but routine testing for C-reactive protein (CRP), fecal calprotectin or food allergies is not recommended. A trial of a low fermentable oligosaccharides, disaccharides, monosaccharides, polyols (FODMAP) diet is suggested, while a gluten-free diet is not. Psyllium, but not wheat bran, supplementation may help reduce symptoms. Alternative therapies such as peppermint oil and probiotics are suggested, while herbal therapies and acupuncture are not. Cognitive behavioural therapy and hypnotherapy are suggested psychological therapies. Among the suggested or recommended pharmacological therapies are antispasmodics, certain antidepressants, eluxadoline, lubiprostone, and linaclotide. Loperamide, cholestyramine and osmotic laxatives are not recommended for overall IBS symptoms. The nature of the IBS symptoms (diarrhea-predominant or constipation-predominant) should be considered in the choice of pharmacological treatments.

**Conclusions:**

Patients with IBS may benefit from a multipronged, individualized approach to treatment, including dietary modifications, psychological and pharmacological therapies.

Irritable bowel syndrome (IBS) is one of the most common gastrointestinal (GI) disorders. It is characterized by recurrent abdominal pain and altered bowel habits (i.e., constipation, diarrhea or both), often with associated bloating (1).

Globally, IBS is estimated to affect about 10% of the general population, but prevalence rates are highly variable (2, 3). From country to country, the prevalence ranges from 1.1% to 45.0% but has been estimated at 12% (95% CI, 7%–17%) generally in North America (2), with a similar prevalence in Canada specifically (4). Rates also vary according to diagnostic criteria (2–5). A large survey, across the United States, United Kingdom and Canada found that the Rome IV (~6%) prevalence rates were significantly lower than Rome III rates (~11%) in all countries (5). Women appear to be affected about 1.5 to two times more often than men, and the prevalence appears to decrease with increasing age (2–5).

The current recommended diagnostic criteria for IBS are the Rome IV criteria: abdominal pain (≥1 day per week for ≥3 months) associated with defecation or a change in bowel habits (1, 6). Irritable bowel syndrome is then subtyped according to the abnormality of stool consistency, including constipation-predominant (IBS-C, >25% hard stools and <25% loose stools), diarrhea-predominant (IBS-D, >25% loose stools and <25% hard stools), mixed bowel habits (IBS-M, >25% loose stools and >25% hard stools); and unclassified (IBS-U <25% loose stools and <25% hard stools) (1). However, the available randomized clinical trials (RCTs) have used a variety of criteria, including Manning, Rome I, II, III, IV or questionnaire-defined. Therefore, for this consensus, IBS was pragmatically defined as abdominal pain associated with change in bowel habit for at least three months, and studies were included if they met this definition, regardless of criteria used. When diagnosing IBS, a full history and relevant examination are recommended, with limited use of diagnostic tests as discussed in the recommendations section of this consensus.

There have been guidelines on the management of IBS (7, 8), but there has been an emergence of new therapies and new RCTs on current therapies because these guidelines have been published. The purpose of this guideline is to critically review the literature relating to diagnostic testing and the psychological and pharmacological treatment of IBS and to develop a consensus on specific recommendations for patients with any IBS, IBS-C or IBS-D.

## METHODS

### Scope and Purpose

These consensus statements focused on specific questions, identified and discussed by the participants, regarding the management of IBS. The development of this clinical practice guideline began in January 2017, with the full consensus group participating in a face-to-face meeting in May 2017; supplementary statements on breath testing were added after the meeting.

### Sources and Searches

The editorial office of the Cochrane Upper Gastrointestinal and Pancreatic Diseases Group at McMaster University performed a systematic literature search of MEDLINE (1946 to March 2017), EMBASE (1980 to March 2017), and CENTRAL (Cochrane Central Register of Controlled Trials). Evidence was first gathered from the most recently published, high-quality systematic reviews (primarily from the American College of Gastroenterology monograph published in 2014 (7), on which PM was a co-author). These meta-analyses were then updated with any further data identified in the literature searches up to March 2017. The updated search strategies are described in Appendix 1 online. Study inclusion criteria were parallel group RCTs (crossover studies were included if the data were available from the first period such that parallel group data could be obtained), using any definition of IBS, including a dichotomous outcome measure relating to global IBS symptom improvement, human studies, and English publications. Further details regarding the search strategies used for preparing the initial consensus statements can be found in Appendix 1 online. Two additional systematic searches (up to May 2017) were performed after the consensus meeting to address statements eight and nine, with key search terms including irritable bowel syndrome, breath tests, and lactose intolerance.

### Review and Grading of Evidence

A methodologist (PM) used the GRADE (Grading of Recommendation Assessment, Development and Evaluation) approach (9) to assess the risk of bias (of individual studies and overall across studies), indirectness, inconsistency, imprecision and other considerations (including publication bias) to determine the overall quality of evidence for each statement. The quality of evidence (QoE) for the individual statements was classified as high, moderate, low or very low as described in GRADE methodology (9, 10) and used in prior Canadian Association of Gastroenterology (CAG) consensus documents (11–16). GRADE assessments were validated by a second, nonparticipating methodologist, then reviewed and agreed upon by voting members of the consensus group at the meeting.

Approved product labeling from government regulatory agencies varies from country to country, and though not ignored, recommendations are based on evidence from the literature and consensus discussion and may not fully reflect the product labeling for a given country.

### Consensus Process

This Canadian consensus group consisted of 12 voting participants with experience in the area of IBS, including the chair (PM), gastroenterologists, general practitioners, a psychiatrist, a psychologist, a patient representative, and the moderator (also a gastroenterologist, WP).

The CAG web-based consensus platform (ECD solutions, Atlanta, Georgia, USA) was used to initiate the consensus process before the face-to-face consensus meeting held in Chicago, Illinois, USA in May 2017. The meeting chair (PM) and the steering committee (CA, GM, CK and the patient-representative [MM]) developed the initial questions. The voting members then used the web-based platform to vote on their level of agreement and provide comments on the questions which were to be used to develop the recommendation statements during the meeting. A summary report of the literature search results was provided to the participants before the meeting.

At the meeting, the methodologist/chair (PM) presented the data and provided the group with a review of the GRADE evaluations which informed the quality of evidence determination for each of the questions. Recommendation statements were developed that were subsequently voted on anonymously via touchpads. A statement achieved consensus and was accepted if ≥75% of participants voted four (agree) or five (strongly agree) on a scale of one to five (with one, two, and three indicating disagree strongly, disagree, and neutral, respectively). Following acceptance of a statement, participants voted on the strength of the recommendation. A level of agreement of ≥75% of participants was needed to classify a statement as ‘strong’ (we recommend); if this threshold was not met, the statement defaulted to ‘conditional’ (we suggest). The strength of the recommendation considered risk-benefit balance, patients’ values and preferences, cost and resource allocation, and the quality of the evidence. Therefore, it was possible for a recommendation to be classified as strong despite having low-quality evidence or conditional despite the existence of high-quality evidence (17). A strong recommendation is indicative of a more broadly applicable statement (‘most patients should receive the recommended course of action’), whereas a conditional recommendation suggests that clinicians should ‘recognize that different choices will be appropriate for different patients and that they must help each patient to arrive at a management decision consistent with her or his values and preferences’ (17).

At the meeting, the group did not reach consensus on three of the initial 31 statements (no recommendation A–C); thus, these statements were rejected. The evidence has been discussed in the text, but the consensus group did not make a recommendation (neither for nor against) offering these treatments to IBS patients.

The initial manuscript was drafted by the meeting chair/methodologist (PM) and was then reviewed and revised by the remaining members of the consensus group. The manuscript was made available to all CAG members for comments for a two-week period before submission for publication, as per CAG policy for all clinical practice guidelines.

In accordance with CAG policy, written disclosures of any potential conflicts of interest for the 24 months before the consensus meeting were provided by all participants and made available to all group members.

### Role of the Funding Sources

Funding for the consensus meeting was provided by unrestricted, arms-length grants to the CAG by Allergan Canada Inc. and Proctor & Gamble Canada. The CAG administered all aspects of the meeting, and the funding sources had no involvement in the process at any point nor were they made aware of any part of the process from the development of search strings and the statements to the drafting and approval of these guidelines.

## RECOMMENDATIONS

The individual recommendation statements are provided and include the GRADE of supporting evidence and the voting results, after which a discussion of the evidence considered for the specific statement is presented. A summary of the recommendation statements is provided in Table 1.

**Table 1.** Summary of consensus recommendations for the management of IBS

DIAGNOSTIC TESTING FOR IBS
**1: We suggest IBS patients have serological testing to exclude celiac disease**. GRADE: Conditional recommendation, low-quality evidence
**2: We recommend AGAINST testing for CRP in IBS patients to exclude inflammatory disorders**. GRADE: Strong recommendation, very low-quality evidence
**3: We recommend AGAINST routine testing for fecal calprotectin in IBS patients to exclude inflammatory disorders**. GRADE: Strong recommendation, very low-quality evidence
**4: We recommend AGAINST IBS patients <50 years of age without alarm features ROUTINELY having a colonoscopy to exclude alternate diagnoses**. GRADE: Strong recommendation, very low-quality evidence
**5: We suggest AGAINST IBS patients <50 years of age with alarm features ROUTINELY having a colonoscopy to exclude alternate diagnoses.** GRADE: Conditional recommendation, very low-quality evidence
**6: We recommend patients with new-onset IBS symptoms at ≥50 years of age have colonoscopy to exclude alternative diagnoses**. GRADE: Strong recommendation, low-quality evidence
**7: We recommend AGAINST IBS patients having food allergy testing to identify triggers of IBS symptoms**. GRADE: Strong recommendation, very low-quality evidence
**8: We recommend AGAINST the routine use of lactose hydrogen breath tests in evaluating IBS patients**. GRADE: Strong recommendation, very low-quality evidence
**9: We recommend AGAINST the routine use of glucose hydrogen breath tests in evaluating IBS patients**. GRADE: Strong recommendation, very low-quality evidence
**DIETARY MODIFICATIONS AND ALTERNATIVE THERAPIES FOR IBS**
**10: We suggest offering IBS patients a low FODMAP diet to reduce IBS symptoms**. GRADE: Conditional recommendation, very low-quality evidence
**11: We suggest AGAINST offering IBS patients a gluten-free diet to reduce IBS symptoms**. GRADE: Conditional recommendation, very low-quality evidence
**12: We suggest AGAINST offering IBS patients wheat bran supplementation to improve IBS symptoms**. GRADE: Conditional recommendation, low-quality evidence
**13: We recommend offering IBS patients psyllium supplementation to improve IBS symptoms**. GRADE: Strong recommendation, moderate-quality evidence
**14: We suggest AGAINST offering herbal remedies to IBS patients to improve IBS symptoms**. GRADE: Conditional recommendation, very low-quality evidence
**15: We recommend AGAINST offering acupuncture to IBS patients to improve IBS symptoms**. GRADE: Strong recommendation, very low-quality evidence
**16: We suggest offering IBS patients peppermint oil to improve IBS symptoms**. GRADE: Conditional recommendation, low-quality evidence
**17: We suggest offering IBS patients probiotics to improve IBS symptoms**. GRADE: Conditional recommendation, low-quality evidence
**PSYCHOLOGICAL THERAPIES FOR IBS**
**18: We suggest offering IBS patients cognitive behavioral therapy to improve IBS symptoms**. GRADE: Conditional recommendation, very low-quality evidence
**19: We suggest offering IBS patients hypnotherapy to improve IBS symptoms**. GRADE: Conditional recommendation, very low-quality evidence
**PHARMACOLOGICAL THERAPIES FOR IBS**
**20: We suggest offering IBS patients certain antispasmodics (such as dicyclomine, hyoscine, pinaverium) to improve IBS symptoms**. GRADE: Conditional recommendation, very low-quality evidence
**21: We recommend offering IBS patients low-dose tricyclic antidepressants to improve IBS symptoms**. GRADE: Strong recommendation, high-quality evidence
**22: We suggest offering IBS patients SSRIs to improve IBS symptoms**. GRADE: Conditional recommendation, moderate-quality evidence
PHARMACOLOGICAL THERAPIES FOR IBS
**23: We suggest AGAINST offering diarrhea-predominant IBS patients continuous loperamide use to improve IBS symptoms.** GRADE: Conditional recommendation, very low-quality evidence
**24: We suggest AGAINST offering diarrhea-predominant IBS patients cholestyramine to improve IBS symptoms**. GRADE: Conditional recommendation, very low-quality evidence
**25: We suggest offering diarrhea-predominant IBS patients eluxadoline to improve IBS symptoms**. GRADE: Conditional recommendation, moderate-quality evidence
**26: We suggest AGAINST offering constipation-predominant IBS patients osmotic laxatives to improve OVERALL IBS symptoms**. GRADE: Conditional recommendation, very low-quality evidence
**27: We suggest AGAINST offering constipation-predominant IBS patients prucalopride to improve OVERALL IBS symptoms**. GRADE: Conditional recommendation, very low-quality evidence
**28: We suggest offering constipation-predominant IBS patients lubiprostone to improve IBS symptoms**. GRADE: Conditional recommendation, moderate-quality evidence
**29: We recommend offering constipation-predominant IBS patients linaclotide to improve IBS symptoms**. GRADE: Strong recommendation, high-quality evidence
**STATEMENTS WITH NO RECOMMENDATIONS**
**No recommendation A:** *The consensus group does not make a recommendation (neither for nor against) offering IBS patients relaxation techniques to improve IBS symptoms.* **No recommendation B:** *The consensus group does not make a recommendation (neither for nor against) offering IBS patients short-term psychodynamic psychotherapy to improve IBS symptoms.* **No recommendation C:** *The consensus group does not make a recommendation (neither for nor against) offering diarrhea-predominant IBS patients one course of rifaximin therapy to improve IBS symptoms.*

*The strength of each recommendation was assigned by the consensus group, per the GRADE system, as strong (‘we recommend . . .’) or conditional (‘we suggest . . .’). A recommendation could be classified as strong despite low quality evidence to support it, or conditional despite the existence of high-quality evidence due to the four components considered in each recommendation (risk:benefit balance, patients’ values and preferences, cost and resource allocation, and quality of evidence).

**Statement 1: We suggest IBS patients have serological testing to exclude celiac disease.**

GRADE: Conditional recommendation, low-quality evidence. Vote: strongly agree, 50%; agree, 50%

**Key evidence:** There was low-quality evidence on the role of celiac testing in IBS from a systematic review of 13 observational studies (including 2021 IBS patients and 2978 controls), which found a pooled prevalence rate for positive celiac antibody tests of 1.63% (95% CI, 0.7–3.0) using tissue transglutiminase (TTG) or endomysial IgA (18). Data from seven case-control studies showed a greater likelihood of having positive celiac antibodies among patients with IBS compared with controls without IBS (odds ratio [OR] 2.94; 95% CI, 1.36–6.35) (18). The quality of evidence for this statement was graded as low due to the observational nature of data.

**Discussion:** The symptoms of celiac disease often overlap with those of IBS, and data suggest that IBS patients are at increased risk of celiac disease (18, 19). There was insufficient data to determine whether the prevalence of celiac disease is higher in patients with IBS-D compared with those with IBS-C or those with alternating symptoms (18). However, the consensus group agreed that testing should be prompted by symptoms suggestive of celiac disease, for example diarrhea-predominant rather than constipation-predominant IBS. In addition, testing for celiac should be performed only once, and if negative, patients do not require a gluten-free diet (GFD).

**Statement 2: We recommend AGAINST testing for CRP in IBS patients to exclude inflammatory disorders.**

GRADE: Strong recommendation, very low-quality evidence. Vote: strongly agree, 67%; agree, 33%

**Key evidence:** There was very-low quality evidence on the role of testing for C-reactive protein (CRP) in IBS patients from a systematic review of biomarker studies, which included four case-control studies (n=224 IBS patients, n=465 inflammatory bowel disease [IBD] patients and n=134 healthy controls) of CRP (20). The main role for CRP is to identify inflammatory disease such as IBD in patients with IBS symptoms; however, the performance of this marker is insufficient to be clinically useful. For example, a CRP of ≤0.5 mg/dL predicted a ≤1% probability of having IBD. Thus, it would appear that CRP has a good negative predictive value, but because the underlying risk of having IBD among the general population with symptoms is also <1%, it is unclear what additional value CRP testing would have in IBS patients. The evidence for this statement was graded as very-low quality due to the observational nature of the data, indirectness and heterogeneity in the studies assessed.

**Discussion:** The consensus group concluded that CRP was unlikely to have value for the diagnosis of IBS or to effectively rule out IBD. Patients with IBS appear to have only a mildly elevated risk of IBD compared with the individuals without IBS, but the absolute risk remains low (see statements four and five). Therefore, CRP testing is likely not warranted on the basis of an IBS diagnosis alone and should be performed only in patients in whom there is a high suspicion of IBD.

**Statement 3: We recommend AGAINST routine testing for fecal calprotectin in IBS patients to exclude inflammatory disorders.**

GRADE: Strong recommendation, very low-quality evidence. Vote: strongly agree, 67%; agree, 33%

**Key evidence:** There was very-low quality evidence on the value of testing for fecal calprotectin (FC) in patients with IBS. The systematic review of biomarkers included eight case-control studies (n=259 IBS patients, n=565 IBD patients and n=238 healthy controls) evaluating FC (20). Neither a low nor high level of FC was predictive of IBS. In comparison with healthy controls, the highest predictive value for IBS was only 18.8% at 280 µg/g (however, this level also predicted a 17.1% chance of IBD). In contrast, a low FC level could exclude IBD (<40 µg/g predicted a ≤1% chance of IBD), and the likelihood of IBD increased with increasing FC levels (1000 μ/g predicted a 78.7% of IBD) (20). The evidence for this statement was graded as very low–quality due to indirectness and the case-control study designs because this design overestimates the diagnostic accuracy of the test.

**Discussion:** Neither high nor low FC levels can completely exclude IBS (20). Although levels <50 µg/g suggest an absence of IBD, levels between 50 and 250 µg/g are equivocal and would not be helpful. In addition, as was the case for CRP, although FC levels are predictive of IBD, the consensus group agreed that because IBS is associated with only a mildly elevated risk of IBD, FC testing should be performed only in patients in whom there is a high suspicion of IBD.

**Statement 4: We recommend AGAINST IBS patients <50 years of age without alarm features ROUTINELY having a colonoscopy to exclude alternate diagnoses.**

GRADE: Strong recommendation, very low-quality evidence. Vote: strongly agree, 92%; agree, 8%

**Statement 5: We suggest AGAINST IBS patients <50 years of age with alarm features ROUTINELY having a colonoscopy to exclude alternate diagnoses.**

GRADE: Conditional recommendation, very low-quality evidence. Vote: strongly agree, 25%; agree, 75%

**Key evidence:** A systematic review was conducted for these consensus guidelines to evaluate the prevalence of other diagnoses (e.g., IBD or microscopic colitis) in IBS patients undergoing colonoscopy. Among patients with IBS symptoms, the rate of IBD diagnosis was 4% (95% CI, 1–9; nine studies, n=5603) (21–29), and the rate of microscopic colitis was 4.5% (95% CI, 2.4–7.3; nine studies, n=3344) (21, 22, 25, 27, 29–33). In patients with IBS-D only, the rate of microscopic colitis was higher at 10% (95% CI, 3–19; six studies, n=841) (23, 29–32, 34). While this data might suggest colonoscopy is useful, there was no age limit in these studies, and all suffered from referral bias. There was significant heterogeneity. In addition, in the majority of studies that included a control group without IBS symptoms, there was no significant difference in the frequency of colonoscopic findings in the IBS group compared with the control groups (21, 22, 24, 27), with the exception of microscopic colitis in females with diarrhea over the age of 45 years (23, 31, 33, 34).

A longitudinal, case-control study using data from a national health insurance database in Taiwan compared the 10-year risk of organic diseases among patients with IBS (n=1225) with age- and sex-matched controls (n=4900) (35). Patients with IBS, irrespective of age, had an increased 10-year risk of being diagnosed with any organic disease (hazard ratio [HR] 1.77; 95% CI, 1.63–1.92; P*<*0.001), microscopic colitis (HR 1.72; 95% CI, 1.58–1.87; P*<*0.001), IBD (HR 1.92; 95% CI, 1.49–2.48; P*<*0.001), or colorectal cancer (CRC) (HR 3.63; 95% CI, 2.54–5.19; P<0.001) compared with controls. Unexpectedly, no cases of celiac disease were reported in the IBS group. Although significantly higher in the IBS group than in the non-IBS group, with the exception of microscopic colitis (59.8% versus 43.6%), the absolute incidence rates of IBD (7% versus 3.8%) and CRC (4.6% versus 1.3%) remained low (35). This study did not stratify by age; therefore, the rates in patients under 50 years could not be determined.

The utility of alarm features in predicting CRC was evaluated in a systematic review of 15 studies (n=19,443) in unselected adults (36). Using a positive likelihood ratio (LR) of 10, which usually signifies a diagnostically useful test (37), most of the included alarm features performed poorly (36). A positive LR was noted for rectal bleeding (unspecified) (LR 1.32’ 95% CI, 1.19–1.47), change in bowel habit (LR 1.29; 95% CI, 1.05–1.59), anemia (LR 1.43; 95% CI, 0.75–2.74), and weight loss (LR 1.96; 95% CI, 1.25–3.08). The only two symptoms with sufficient specificity to rule in the diagnosis of CRC (therefore suggesting that colonoscopy would be appropriate) were abdominal mass (97%; 95% CI, 96–98) and dark red rectal bleeding (96%; 95% CI, 93–98).

**Discussion:** Data were conflicting, and while some evidence suggested that IBS patients are at increased risk for organic disease over the long-term compared with individuals in the general population, absolute rates remain low.

With respect to CRC, the risk is low in the general population <50 years of age (38), and IBS is not a recognized risk factor for CRC (39, 40). There appears to be little or no evidence that IBS increases the risk of CRC over the short-term compared with the general population (41, 42), with the exception of the study from Taiwan that suggested a 3.6 times higher 10-year risk in the IBS group compared with the non-IBS group (35).

Data in the general population suggested that the diagnostic accuracy of alarm symptoms for CRC was poor. Alarm features are usually defined as vomiting, gastrointestinal bleeding, abdominal mass, dysphagia, unexplained weight loss and anemia (43). Only abdominal mass and dark red rectal bleeding were associated with an increased risk of CRC (36).

An observational study of the predictive value of alarm features, specifically in IBS patients, did suggest a significantly higher prevalence of organic diseases (including Crohn’s disease, celiac disease, and microscopic colitis) among those with alarm features compared with those without (27.7% versus 15.4%, P=0.002) (44). There was a trend toward a higher rate of CRC among IBS patients with alarm features, but this was not significant. The likelihood of organic disease increased with increasing numbers of alarm features. Importantly, the prevalence of organic diseases was significantly higher in patients with IBS-D compared with those with IBS-C.

Finally, data do not support the idea that patients may be reassured by a normal colonoscopy. A retrospective study in patients with IBS aged <50 years found no difference in the proportions of patients that were ‘reassured’ (defined as a negative response to the question ‘Do you think something serious is wrong with your body?’) by negative colonoscopy compared with no colonoscopy (45)

Therefore, the consensus group concluded that routine colonoscopy is generally not warranted in IBS patients <50 years of age, and alarm symptoms do not appear to increase the risk of CRC sufficiently to warrant routine colonoscopy. However, clinical judgement is important, and colonoscopy and flexible sigmoidoscopy would continue to play a role to investigate specific indications such as a high clinical suspicion of IBD or microscopic colitis or for patients with a combination of alarm features or a pronounced alarm feature such as dramatic weight loss.

**Statement 6: We recommend patients with new-onset IBS symptoms at ≥50 years of age have colonoscopy to exclude alternative diagnoses.**

GRADE: Strong recommendation, low-quality evidence. Vote: strongly agree, 50%; agree, 50%

**Key evidence:** The support for this statement is extrapolated from evidence-based guidelines on screening for CRC (39, 40, 46). Colonoscopy is regarded as the gold-standard screening test for CRC, and while some guidelines (40) do not recommend this approach in the average-risk population, the opportunistic screening of patients ≥50 years of age with recently onset IBS symptoms that have not had investigation within five years would involve fewer individuals than a general population screening program. Therefore, this would likely be less costly and the benefits would be likely to outweigh the risks. There is moderate-quality evidence that flexible sigmoidoscopy is associated with a decreased incidence of CRC, which when extrapolated to colonoscopy is reduced to low quality because of indirectness (46). Additional, low-quality, observational data have shown that screening colonoscopy in individuals at average risk can reduce the relative risk of CRC incidence but not CRC mortality (47).

**Discussion:** An estimated 50% of patients with IBS report having first had symptoms before the age of 35 years (48), and the odds of IBS in individuals over 50 years were significantly lower than in those younger than 50 years (OR, 0.75; 95% CI, 0.62–0.92) (2). Therefore, the new onset of IBS symptoms in older patients may warrant colonoscopy to exclude other diagnoses (e.g., CRC or IBD).

Guidelines recommend that individuals 50 years or above at average risk for CRC undergo screening with fecal occult blood testing or colonoscopy (39, 40). As mentioned (see statements four and five), there appears to be little evidence that IBS symptoms alone increase the risk of CRC (41, 42); therefore, the consensus group agreed that adults 50 years or older with an established history of IBS should be screened for CRC according to average risk guidelines (39, 40).

The consensus group emphasized that colonoscopy is just one method of screening for CRC, and patients should be made aware and educated about the efficacy of fecal immunochemical tests (FITs) in reducing CRC incidence and mortality (39). This is particularly important in light of the greater resource requirements associated with colonoscopy (40). Patient should be engaged in informed decision-making about screening, and preferences for colonoscopy or FIT should be considered (39).

**Statement 7: We recommend AGAINST IBS patients undergoing food allergy testing to identify triggers of IBS symptoms.**

GRADE: Strong recommendation, very low-quality evidence. Vote: strongly agree, 100%

**Key evidence:** The efficacy of food allergy testing has been evaluated in one RCT, in which 150 patients were randomized to exclude all foods that they were intolerant of according to an IgG antibody test or to a sham diet where patients were asked to avoid the same number of foods to which they exhibited IgG antibodies but not those particular foods (49). The trial had an unclear risk of bias. At 12 weeks, a greater proportion of patients in the active intervention group reported a ‘significantly improved’ global impact score versus those in the sham diet group (28% versus 17%, number needed to treat [NNT] 9). But this was not statistically significant (P=0.14). This was higher among patients who fully adhered to their diets (54.1% versus 15%, NNT 2.5).

**Discussion:** Food adverse reactions include food allergy and food intolerance. While food allergies are mediated by immunologic reactions, food intolerance is not and may be due to factors within foods, such as chemical agents (e.g., caffeine or tyramine), enzyme deficiency of the host (e.g., lactase deficiency) or idiosyncratic responses related to an unknown mechanism (50, 51). Although patients with IBS self-report food allergies more often than the general population, evidence suggests that true food allergies are relatively uncommon. True food allergies are reported in about 1% to 4% of adults in the general population, with 50% to 90% of presumed food allergies being food intolerances (51). In a study in patients with GI symptoms, food allergy was confirmed by endoscopic allergen provocation or elimination diet and rechallenged in only 3.2% of patients, despite 32% of patients with GI symptoms reporting adverse reactions to food (52). Another study found no difference in the frequency of positive skin prick tests in IBS patients with and without self-reported adverse reactions to food, and symptoms correlated with the foods identified by allergy testing in only 14% of cases (53).

In contrast, food triggers are common, with more than half of IBS patients having self-reported food intolerances (54, 55). The most common food triggers include those with incompletely absorbed carbohydrates (e.g., dairy products, beans/lentils, apple and flour), foods rich in biogenic amines (e.g., wine/beer, salami and cheese), histamine-releasing foods (e.g., milk, wine/beer and pork), and fried and fatty foods (54, 56).

The consensus group concluded that true food allergies are likely to be rare in IBS patients, and there are little data to support the efficacy of avoiding foods as identified by allergy testing. Therefore, they strongly recommended against IBS patients undergoing allergy testing and encouraged patient education to discourage this practice. This suggestion does not preclude encouraging patients to avoid specific foods that have been associated with symptom exacerbations, but allergy testing is unlikely to provide any additional benefit.

**Statement 8: We recommend AGAINST the routine use of lactose hydrogen breath tests in evaluating IBS patients.**

GRADE: Strong recommendation, very low-quality evidence. Vote: strongly agree, 67%; agree, 25%; neutral, 8%

**Key evidence**: A systematic review was conducted for these consensus guidelines to evaluate the prevalence of lactose malabsorption in patients with IBS. In a meta-analysis of 34 case series including 9041 patients with all subtypes of IBS evaluated with a lactose H_2_ breath test, the prevalence of lactose malabsorption was 47% (95% CI, 41%‒53%) with significant heterogeneity between studies. The prevalence was 50% (95% CI, 43%‒56%) in European studies, 21% (95% CI, 14%‒29%) in US studies, and 56% (95% CI, 43%‒69%) in South Asian studies. Heterogeneity persisted in these subanalyses, suggesting that the variability in results could not be explained by different genetic populations with different underlying risks of lactase deficiency but was likely due to variations in patient selection, doses of lactose used and cutoffs to define malabsorption.

**Figure 1.**
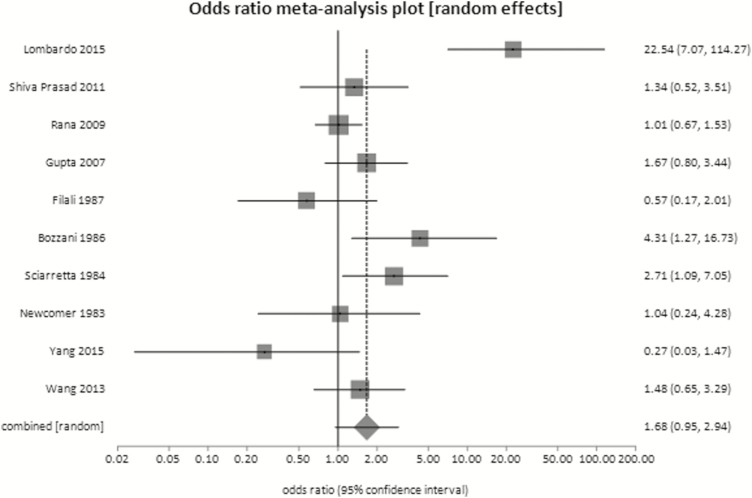
Proportion of subjects with lactose intolerance in IBS patients and healthy volunteers

In 10 case control studies, including 2008 subjects, there was no significant difference in the prevalence of lactose malabsorption in IBS patients compared with controls with no GI symptoms (OR 1.68; 95% CI, 0.95‒2.94, P*=*0.07) (Figure 1). Again, there was significant heterogeneity between studies.

**Discussion**: While lactose malabsorption is common, there appears to be no significant difference in its prevalence in patients with IBS compared with the general population. Furthermore, the few studies that have assessed the efficacy of lactose avoidance in improving symptoms in IBS patients show conflicting results (57–62).

The data do not exclude lactose malabsorption as the explanation for IBS symptoms in a minority of patients, but they do not support the routine use of lactose breath testing in IBS patients.

**Statement 9: We recommend AGAINST the routine use of glucose hydrogen breath tests in evaluating IBS patients.**

GRADE: Strong recommendation, very low-quality evidence. Vote: strongly agree, 50%; agree, 50%

**Key evidence:** A systematic review of studies evaluating the prevalence of small intestinal bacterial overgrowth (SIBO) in patients with IBS was conducted for these consensus guidelines. In a meta-analysis of 24 case series, including 2698 patients with all subtypes of IBS evaluated with a glucose H_2_ breath test, the prevalence of SIBO was 25% (95% CI, 19%‒32%) with significant heterogeneity between studies and use of a variety of nonvalidated cutoffs. This analysis excluded studies evaluating the lactulose H_2_ breath test, which may be less specific (63).

**Figure 2.**
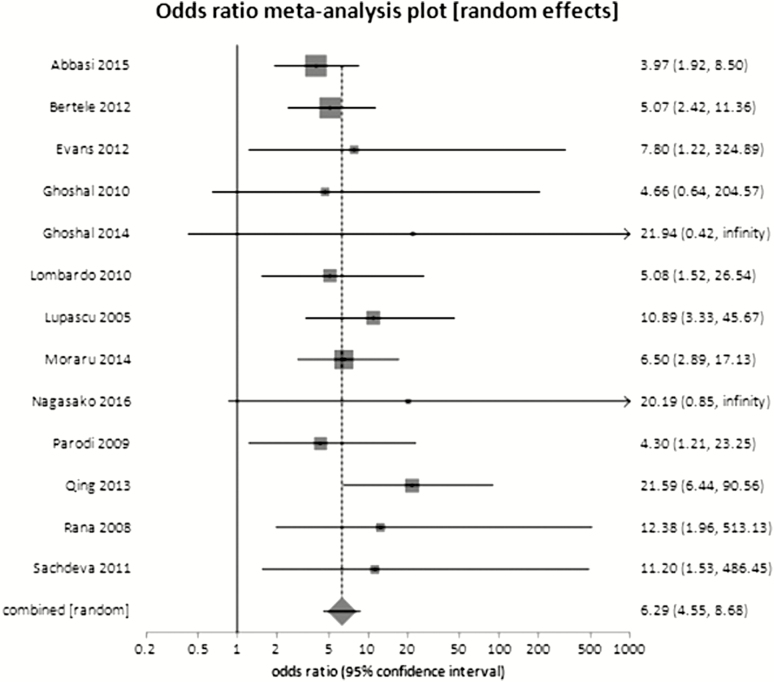
Proportion of SIBO in IBS patients and healthy volunteers

In 13 case-control studies, including a total of 2682 participants, there was a statistically significant difference in the prevalence of SIBO in patients with IBS compared with controls without GI symptoms (OR 6.29; 95% CI, 4.55‒8.68) (Figure 2). There was no significant heterogeneity between studies.

**Discussion:** Although a strong difference in the prevalence of SIBO was found between patients with IBS and healthy controls, clinicians do not require a test to differentiate between those with IBS and those who are asymptomatic. Studies have rarely evaluated other control groups, but those that did found that SIBO was either just as or more prevalent in those with functional diarrhea (63) and functional dyspepsia (64), with the latter finding not explained by proton pump inhibitor use.

Furthermore, there is a paucity of studies of antibiotic therapy for improving symptoms in patients with a positive glucose breath test (versus negative breath test), and most have focused on only those that are breath test positive (65, 66). This approach does not define the value of a positive test.

The consensus group concluded that although SIBO may be an explanation for IBS symptoms for some patients, the data do not support the routine use of glucose breath testing in IBS patients.

**Statement 10: We suggest offering IBS patients a low FODMAP diet to reduce IBS symptoms.**

GRADE: Conditional recommendation, very low-quality evidence. Vote: strongly agree, 27%; agree, 64%; neutral, 9%

**Key evidence:** Evidence for a low FODMAP (fermentable oligosaccharides, disaccharides, monosaccharides, polyols) diet was available from four RCTs including 248 patients with IBS (67–71). Comparators were an alternative diet (two studies), a high FODMAP diet (one study), or an usual diet (one study). Overall, there was a trend toward an improvement in IBS symptoms with a low FODMAP diet (risk ratio [RR] of IBS symptoms not improving 0.67; 95% CI, 0.45–1.00; P*=*0.05). The two trials (n=167) with adequate blinding comparing a low FODMAP diet to an alternative diet showed no benefit (RR 0.89; 95% CI, 0.68–1.17; P*=*0.84) (67–69). Preliminary data from a large RCT (n=104) reported significant benefit with the low FODMAP diet compared with the sham diet; however, this study was only available in abstract form (72). GRADE analysis found all trials to be at high-risk of bias, with significant clinical and statistical heterogeneity between studies.

**Discussion:** The interest in a low FODMAP diet stems from the fact that FODMAPs are carbohydrates that are largely indigestible in the small intestine because of the absence of suitable hydrolase enzymes or incomplete absorption (73). This leads to an increase in fluid in the small bowel, which may be one of the underlying mechanisms for diarrhea in IBS. In addition, fermentation of FODMAPs by colonic microbiota results in production of gas, short chain fatty acids, and possibly other metabolites (73–76).

There was a trend toward a beneficial effect of low FODMAP diet, but the data were very low in quality. Patients will often want to explore dietary changes (77), and the low FODMAP appears to be the most studied and perhaps the most likely to improve overall symptoms (see also statement 11). Most studies also showed significant improvements in individual symptoms of abdominal pain, bloating, frequency and urgency (68–71). Increases in dietary FODMAP content were associated with increasing symptoms, demonstrating the importance of adherence to the diet (70).

The low FODMAP diet has potential drawbacks. Of concern is the fact that the low FODMAP diet can have a substantial effect on the colonic microbiota. Studies have shown a reduction in the relative proportion of Bifidobacteria and other changes that may negatively affect gastrointestinal health (70–72, 78). The included RCTs were all short-term (three to four weeks) (68–71), and long-term use is generally not recommended due to the risk of dietary inadequacy related to the exclusion of many nutrient-rich foods (73). From a patient’s perspective, the low FODMAP diet can be difficult to adhere to, costly, and restricting for social events such as dining out (73, 79).

The consensus group concluded that there were some data to suggest that a low FODMAP diet may be helpful for some patients. However, if a low-FODMAP diet is suggested, it should be implemented under the guidance of a dietician, and a strict diet should be implemented for as short a term as possible (e.g., four weeks).

**Statement 11: We suggest AGAINST offering IBS patients a gluten-free diet to reduce IBS symptoms.**

GRADE: Conditional recommendation, very low-quality evidence. Vote: strongly agree, 64%; agree, 36%

**Key evidence:** Two RCTs evaluated a gluten-free diet in 111 patients with IBS in whom celiac disease had been rigorously excluded (67, 80, 81). In these rechallenge trials, IBS patients who reported symptom control with a gluten-free diet were then randomized to a gluten challenge or continued a gluten-free diet. This provides indirect data because withdrawing a significant food group from the diet and then introducing it may induce symptoms even in an individual without IBS. In the pooled analysis, there was no statistically significant impact on IBS symptoms in the gluten challenge versus gluten-free diet groups (RR 0.46; 95% CI, 0.16–1.28; P*=*0.14), with significant heterogeneity between studies. A randomized trial suggested that the benefit of a gluten-free diet in patients without celiac disease may relate to the accompanying reduction in FODMAPs (82).

**Discussion:** Nearly two-thirds of IBS patients have reported that their GI symptoms were related to meals (56). Patients commonly associate certain foods with exacerbations of their symptoms, and more than one-half have self-reported food intolerances (54 ,^55^, ^77^). As a result, patients will often try to explore dietary modifications to relieve their symptoms (77).

Perhaps based on the role of gluten in the pathogenesis of celiac disease, gluten has become associated with gastrointestinal symptoms. Conversely, the gluten-free diet is perceived as healthier and has become increasingly popular with the general population (83). However, a gluten-free diet overall is not necessarily healthier because it is associated with high sugar intake and low fibre and mineral intake (84). In addition, gluten-free foods can be difficult to obtain and are more expensive than their gluten-containing counterparts (85).

The consensus group suggested against a gluten-free diet, concluding that it has an uncertain effect on IBS symptoms and places an unnecessary burden on IBS patients.

**Statement 12: We suggest AGAINST offering IBS patients wheat bran supplementation to improve IBS symptoms.**

GRADE: Conditional recommendation, low-quality evidence. Vote: strongly agree, 50%; agree, 50%

**Statement 13: We recommend offering IBS patients psyllium supplementation to improve IBS symptoms.**

GRADE: Strong recommendation, moderate-quality evidence. Vote: strongly agree, 50%; agree, 50%

**Key evidence:** For this consensus, a prior systematic review and meta-analysis (86) was updated with one additional RCT (87) for a total of 15 RCTs (n=946). Most trials did not differentiate between patients according to IBS subtype, and few used Rome criteria to diagnose IBS. Risk of bias was unclear in the majority of studies.

Overall, there was a statistically significant effect in favour of fibre supplementation versus placebo or no treatment (RR of IBS not improving 0.87; 95% CI, 0.80–0.94; *P=*0.0003). Bran (six studies, n=411) had no significant effect on treatment of IBS (RR of IBS not improving 0.90; 95% CI, 0.79–1.03; *P=*0.14); however, ispaghula husk (psyllium) (seven studies, n=499) was effective (RR of IBS not improving 0.83; 95% CI, 0.73–0.94; P*=*0.005; NNT 7; 95% CI, 4–25).

In the updated meta-analysis (seven studies, n=606), there was no increase in overall adverse events with fibre compared with placebo (36.6% versus 25.1%; RR 1.06; 95% CI, 0.92–1.22). There were insufficient data from individual studies to assess adverse events according to fibre type.

**Discussion:** The evidence suggests that only soluble (e.g., ispaghula husk/psyllium) but not insoluble (e.g., wheat bran) fibre had a significant effect for the treatment of IBS symptoms. It has been suggested that insoluble fibre may exacerbate symptoms (7). Psyllium has been shown to improve both constipation and diarrhea (88). The mechanisms are unlikely to be solely related to bulking of stool and may also include alterations in the production of gaseous fermentation products and changes to the composition of the gut microbiome (86, 88).

While increasing fibre content in diet may also be helpful, this is more difficult to regulate and was not studied in the RCTs, which all used fibre supplements. Foods high in soluble fibre include oats, barley, flaxseeds, oranges, carrots, beans/legumes, and those high in insoluble fibre include wheat bran, whole grains, some vegetables (e.g., broccoli, cabbage), and fruits with skins (e.g., apples, grapes) (55).

Based on the evidence for efficacy and safety, the consensus group strongly recommended soluble fibre supplementation as a low-cost, safe treatment option that is acceptable to patients and has moderate-quality evidence that it improves IBS symptoms.

**Statement 14: We suggest AGAINST offering herbal remedies to IBS patients to improve IBS symptoms.**

GRADE: Conditional recommendation, very low-quality evidence. Vote: strongly agree, 73%; agree, 18%; neutral, 9%

**Key evidence:** Evidence for herbal remedies in IBS was available from three systematic reviews (89–91). One systematic review of 72 RCTs concluded that traditional Chinese medicine (TCM) combined with conventional Western medicine improved IBS symptoms (RR 1.21; 95% CI, 1.18–1.24) compared with Western medicine alone using indirect comparisons. However, the authors noted the methodological quality of the included RCTs was very low (89). A second systematic review including 27 studies on a wide variety of herbal therapies found the most evidence for efficacy in IBS for essential oil of Mentha piperita and the compound preparation STW 5, a formula containing hydroethanolic extract of nine herbs. Aloe vera, *Curcuma xanthorriza* and *Fumaria officinalis* showed no benefit in IBS. However, the authors did not synthesize the results from different preparations and were unable to reach a definitive conclusion as to the value of herbal therapies in IBS (90).

The third systematic review evaluated 22 RCTs including 25 different TCM or Western herbal therapies compared with placebo or conventional medicines. Eight studies (using nine herbals) showed global improvement of IBS symptoms, four studies (using three herbals) found efficacy in IBS-D, and two studies (using two herbals) showed efficacy in IBS-C. However, 18 of 22 trials were determined to be of poor quality, and there was evidence of publication bias (91).

**Discussion:** Overall, the evidence for herbal treatments, most of which is derived from studies of TCMs, is very low-quality. Many herbal products are not regulated, and the amount of ‘active ingredient’ can vary among the different products or batches of products. Products that are licensed by Health Canada’s Natural and Non-prescription Health Products Directorate (NNHPD) include an eight-digit natural product number (NPN) or homeopathic medicine number (DIN-HM) on the label. In addition, although the published studies report low rates of adverse events, herbal treatments are not without side effects.

The consensus group concluded that while some studies suggest a benefit of some herbal therapies, there is insufficient evidence for any particular herbal product that would warrant recommending any individual therapy. Conversely, it cannot be ruled out that some products may be effective, and more high-quality studies are needed.

**Statement 15: We recommend AGAINST offering acupuncture to IBS patients to improve IBS symptoms.**

GRADE: Strong recommendation, very low-quality evidence. Vote: strongly agree, 64%; agree, 36%

**Key evidence:** A Cochrane systematic review identified six RCTs evaluating acupuncture in IBS. Pooled results from the only two trials providing data for the dichotomous outcome (the proportion of responders with clinically recognized improvement in symptoms) showed no statistically significant effect of acupuncture versus sham acupuncture (RR 1.28; 95% CI, 0.83–1.98; n=109) (92). However, most of the trials were of poor quality and were heterogeneous in terms of interventions, controls and outcomes measured.

An update to the Cochrane systematic review included 17 RCTs (n=1806), but this review did not fulfill our inclusion criteria because it provided no dichotomous data for the acupuncture versus sham comparisons. However, there was no evidence of an improvement with acupuncture relative to sham (placebo) acupuncture for symptom severity (standard mean difference [SMD] −0.11; 95% CI, −0.35 to 0.13; four RCTs; 281 patients) or quality of life (SMD −0.03; 95% CI, −0.27 to 0.22; three RCTs; 253 patients) (93). The overall quality of the evidence in the sham controlled trials was rated moderate due to sparse data.

The proportion of patients with symptom improvement was significantly higher in the acupuncture group compared with pharmacological therapy (84% versus 63%; RR 1.28; 95% CI, 1.12 to 1.45; five studies; 449 patients) and compared with no specific therapy (63% versus 34%; RR 2.11; 95% CI, 1.18 to 3.79; two studies, 181 patients). The overall quality of evidence for this outcome was low due to a high risk of bias (no blinding) and sparse data (93).

Therefore, the current GRADE assessment concluded that there was very low-quality evidence suggesting that there were no benefits of acupuncture relative to a credible sham acupuncture control for proportion of responders or IBS symptom severity (92).

**Discussion:** Data did not show a significant benefit of acupuncture compared with sham acupuncture treatments in improving IBS symptoms overall. A recent RCT that postdated the search window for this consensus provided further support for a lack of efficacy (94).

Data are very low-quality, and it is unknown whether level of therapist training or different acupuncture techniques would impact results. Although generally well tolerated, adverse events of acupuncture can include bleeding, pain and aggravation of symptoms, and rare, serious complications can include tissue or nerve injury and infections (95–97).

**Statement 16: We suggest offering IBS patients peppermint oil to improve IBS symptoms.**

GRADE: Conditional recommendation, low-quality evidence. Vote: strongly agree, 17%; agree, 83%

**Key evidence:** For this consensus, a prior systematic review and meta-analysis (98) was updated with three additional RCTs (99–101) for a total of seven studies (n=634). Only three RCTs differentiated IBS patient types. Risk of bias was unclear in five of the studies and low in the remaining two studies. Rome criteria (II or III) were used to define IBS in five trials.

**Figure 3.**
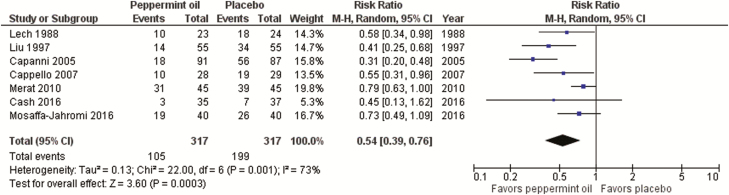
Forest plot of RR of IBS not improving in RCTs of peppermint oil versus placebo in IBS

There was a statistically significant effect in favour of peppermint oil compared with placebo (RR of IBS not improving 0.54; 95% CI, 0.39–0.76; P*=*0.0003; NNT 4; 95% CI, 3–6) (Figure 3); however, there was significant heterogeneity between results and concerns regarding blinding.

Data on overall adverse events were provided in six of the RCTs. The pooled incidence of adverse events was not significantly greater among patients who received peppermint oil versus those who received placebo (11.4% versus 6.0%; RR 1.90; 95% CI, 0.81–4.48).

**Discussion:** While the evidence for peppermint oil is of low quality, results are consistently favourable. However, results should consider that double-blinding may be an issue in these trials because of the menthol breath smell with some products. Administration of peppermint oil can be associated with heartburn and nausea (102), but this may potentially be reduced by encapsulated forms of the product (99, 100).

Based on the evidence suggesting efficacy and safety and the comparatively low cost of the intervention, the consensus group conditionally suggested the use of peppermint oil as a treatment option that may be helpful for patients with IBS.

**Statement 17: We suggest offering IBS patients probiotics to improve IBS symptoms.**

GRADE: Conditional recommendation, low-quality evidence. Vote: strongly agree, 25%; agree, 67%; neutral, 8%

**Key evidence:** For this consensus, a prior systematic review and meta-analysis (86) was updated with 16 additional trials (103–118) for a total of 51 RCTs (n=5448) (119). Risk of bias was unclear in 27 studies and low in 24 studies, and Rome criteria (II or III) were used to define IBS in the majority of trials. Dichotomous outcomes were available from 35 RCTs (n=4306).

Overall, probiotics were statistically significantly superior to placebo (RR of IBS not improving 0.81; 95% CI, 0.74–0.89; P*<*0.00001; NNT 7; 95% CI, 5–12.5) (Table 2). There was significant heterogeneity between studies. Subanalyses according to type of probiotic demonstrated significant effects only for combination probiotics (20 studies; RR 0.79; 95% CI, 0.69–0.92; P*=*0.002), *Escherichia* (two studies; RR 0.86; 95% CI, 0.79–0.93; P*=*0.0003), and *Streptococcus* (one study; RR 0.72; 95% CI, 0.53–0.99; P*=*0.04) (Table 2). No significant benefit was seen with *Lactobacillus* (eight studies; RR 0.82; 95% CI, 0.63–1.06; P*=*0.13), *Saccharomyces boulardii* (two studies; RR 0.92; 95% CI, 0.82–1.03; P*=*0.14), or *Bifidobacterium* (two studies; RR 0.71; 95% CI, 0.44–1.16; P*=*0.18).

**Table 2.** Summary of risk ratios with probiotics versus placebo in IBS

Treatment, # of trials (n)	RR (95% CI); p-value
Probiotics overall, 35 trials (n=4306)	0.81 (0.74–0.89); P*<*0.00001
Combination probiotics, 20 trials (n=1878)	0.79 (0.69–0.92); P*=*0.002
*Lactobacillus*, 8 trials (n=893)	0.82 (0.63–1.06); P*=*0.13
*Saccharomyces boulardii*, 2 trials (n=579)	0.92 (0.82–1.03); P*=*0.14
*Bifidobacterium*, 2 trials (n=484)	0.71 (0.44–1.16); P=0.18
*Escherichia*, 2 trials (n=418)	0.86 (0.79–0.93); P=0.0003
*Streptococcus*, 1 trial (n=54)	0.72 (0.53–0.99); P*=*0.04

Overall probiotics had a statistically significant effect in reducing global symptom or abdominal pain scores (33 studies; SMD –0.21; 95% CI, –0.31 to –0.10; P*=*0.0001), bloating scores (24 studies; SMD –0.13; 95% CI, –0.24 to –0.02; P*=*0.02) and flatulence scores (11 studies; SMD –0.23; 95% CI, –0.38 to –0.08; P*=*0.003) but not urgency scores (8 studies; SMD –0.11; 95% CI, –0.26 to 0.05) compared with placebo.

Data on overall adverse events were reported in 36 of the RCTs. The pooled incidence of adverse events was not significantly greater among patients who received probiotics versus those who received placebo (19.4% versus 17.0%; RR 1.09; 95% CI, 0.91–1.29).

**Discussion:** Probiotics overall and combination products have demonstrated efficacy in improving IBS symptoms. However, trials assessed a variety of combination and specific-species products (but widely variable strains). Significant benefits were seen with combination products (most of which contain *Lactobacillus* but different species) and a trend but no significant benefit with single-species *Lactobacillus* products.

Similar to herbal products, many probiotic products are not regulated. They may have limited shelf life, making the content of live bacteria questionable. Probiotic products can be an economic burden, and patients should be encouraged to select products that are licensed by Health Canada’s NNHPD.

The consensus group concluded that while studies suggest benefits with probiotic therapies overall, and with combination probiotics, there is insufficient evidence for any particular species. Although some products may be helpful, others may not. Therefore, if probiotics are to be used, the consensus group suggested a limited therapeutic trial (e.g., one month). Probiotics can be expensive for patients but are often viewed as a more ‘natural’, low-risk approach to improving IBS symptoms (120).

**Statement 18: We suggest offering IBS patients cognitive behavioural therapy to improve IBS symptoms.**

GRADE: Conditional recommendation, very low-quality evidence. Vote: strongly agree, 17%; agree, 83%

**Statement 19: We suggest offering IBS patients hypnotherapy to improve IBS symptoms.**

GRADE: Conditional recommendation, very low-quality evidence. Vote: agree, 83%; neutral, 17%

*No recommendation A: The consensus group does not make a recommendation (neither for nor against) offering IBS patients relaxation techniques to improve IBS symptoms.*

*No recommendation B: The consensus group does not make a recommendation (neither for nor against) offering IBS patients short-term psychodynamic psychotherapy to improve IBS symptoms.*

**Key evidence:** For this consensus, a prior systematic review and meta-analysis (121) were updated with three new RCTs (122–124) for a total of 35 RCTs (n=2381) (125). The quality of the trials was generally poor, with small sample sizes, lack of blinding and high-risk of bias. About two-thirds of the trials used Rome criteria (I, II or III) to diagnose IBS.

The RCTs evaluated 11 different therapy approaches, and overall, psychological therapy was statistically significantly superior to placebo (35 studies; RR of IBS not improving 0.69; 95% CI, 0.62–0.77; NNT 4; 95% CI, 3–5) (125). There was significant heterogeneity between studies.

Subanalyses according to type of psychological therapy demonstrated significant effects for cognitive behavioural therapy (CBT) (nine studies; RR 0.60; 95% CI, 0.44–0.83; NNT 3; 95% CI, 2–6), hypnotherapy (four studies; RR 0.74; 95% CI, 0.63–0.87; NNT 4; 95% CI, 3–8), multicomponent psychological therapy (five studies; RR 0.72; 95% CI, 0.62–0.83; NNT 4; 95% CI, 3–7) and dynamic psychotherapy (two studies: RR 0.60; 95% CI, 0.39–0.93; NNT 3; 95% CI, 2–25). Multi-component psychological therapy administered via the telephone and contingency management were each studied in 1 trial, and each appeared to have beneficial effects.

No significant benefit was seen with relaxation therapy (seven studies: RR 0.78; 95% CI, 0.62–1.00), stress management (three studies; RR 0.68; 95% CI, 0.39–1.20), self-administered CBT (three studies; RR 0.53; 95% CI, 0.17–1.66), mindfulness meditation training (two studies; RR 0.78; 95% CI, 0.44–1.41) or CBT delivered via the internet (two studies; RR 0.75; 95% CI, 0.48–1.17).

In the four trials (126–129) (n=233) that used ‘sham’ or ‘control’ psychological interventions, there was a statistically significant effect of ‘active’ psychological therapy (RR 0.56; 95% CI, 0.38–0.84; NNT 3; 95% CI, 2–9).

Adverse event data were poorly reported in the individual RCTs, precluding any meaningful analysis.

**Discussion:** There was evidence to suggest that psychological therapies overall, as well as CBT, hypnotherapy, and multi-component psychological therapies can be effective for the management of IBS symptoms. However, the data were of very low quality, with evidence of publication bias or other small study effects that may result in overestimates of the effects of psychological therapies. The majority of studies were small, the control group was often usual care or wait list, and blinding is generally not feasible.

Cognitive behavioural therapy was the most widely studied psychological therapy and was found to be effective. Therapist-administered CBT appears to be necessary because internet-based, self-administered or minimal contact CBT did not have significant benefits overall. In small studies, gut-directed hypnotherapy was also found to be effective overall. The mechanism of action is not simply through improvement of distress or mental health symptoms, as the largest effect sizes were found for gastrointestinal symptoms (130). The consensus group concluded that psychotherapy with CBT or hypnotherapy may be effective for some patients, and while it was suggested as a management option, it was recognized that in clinical practice, availability can be a problem.

Relaxation techniques are generally elements of other types of psychological therapies, such as CBT. Clinical trial results with relaxation therapy only were inconsistent and may depend on the techniques that were used. Relaxation therapy was not statistically significantly different than control, but the 95% confidence intervals did include a clinically important benefit. However, because of the inconsistent results, the consensus group could not make a recommendation for or against this option. Some consensus participants had concerns that if a patient fails relaxation therapy, they may not be willing to try another more effective psychotherapeutic option.

Although evidence suggested significant effects for dynamic psychotherapy, the findings of the two studies came from the same lab, the therapy can be difficult to replicate, and the data do not reflect techniques commonly used in current clinical practice. The studies were short-term (e.g., eight sessions), while in clinical practice, this type of psychotherapy is usually administered over a longer period. Unlike therapies such as CBT that are manualized with a specific protocol, psychodynamic therapies are less structured. The consensus group concluded that the data may not reflect a psychotherapy that is clinically available and were unable to make a recommendation for or against this option.

Overall, the consensus group concluded that while access in Canada may be a problem, psychological therapies—particularly CBT or hypnotherapy—may be a beneficial treatment option for some patients.

**Statement 20: We suggest offering IBS patients certain antispasmodics (such as dicyclomine, hyoscine, pinaverium) to improve IBS symptoms.**

GRADE: Conditional recommendation, very low-quality evidence. Vote: strongly agree, 8%; agree, 75%; neutral, 17%

**Key evidence:** For this consensus, a prior systematic review and meta-analysis (98), was updated with three new studies (131–133) for a total of 26 RCTs (n=2811). The quality of the trials was generally poor with the majority having small sample sizes and high risk of bias. Most trials pre-dated Rome criteria, and the majority used author-defined criteria to diagnose IBS. The majority of trials did not differentiate between the type of IBS patients.

The trials evaluated a total of 13 different antispasmodics, and overall antispasmodic therapy was significantly superior to placebo (RR of IBS symptoms not improving 0.65; 95% CI, 0.56–0.76; *P<*0.00001; NNT 5; 95% CI, 4–8). There was significant heterogeneity between studies.

Subanalyses of individual antispasmodics demonstrated significant benefits compared with placebo for otilonium (5 studies; RR of IBS not improving 0.70; 95% CI, 0.54–0.90; *P=*0.005; NNT 5; 95% CI, 4–11), pinaverium bromide (4 studies; RR 0.56; 95% CI, 0.38–0.82; *P=*0.003; NNT 4; 95% CI, 3–6), hyoscine bromide (three studies; RR 0.63; 95% CI, 0.51–0.78; *P<*0.0001; NNT 3; 95% CI, 2–25), cimetropium bromide (three studies, RR 0.38; 95% CI, 0.20–0.71; *P=*0.002; NNT 3; 95% CI, 2–12.5), drotaverine (2 studies; RR 0.31; 95% CI, 0.19–0.50; *P<*0.00001; NNT 2; 95% CI, 2–3), and dicyclomine (dicycloverine) hydrochloride (1 study, RR 0.65; 95% CI, 0.45–0.95; *P=*0.02; NNT 4; 95% CI, 2–25). Trimebutine (three studies), mebeverine, pirenzipine, alverine, rociverine, prifinium, and propinox (1 study each) did not have a statistically significant effect on IBS symptoms.

Adverse event data were provided in 17 of the RCTs. The pooled incidence of adverse events was significantly greater among patients who received antispasmodics versus those who received placebo (15.3% versus 9.5%; RR 1.60; 95% CI, 1.15–2.21; number needed to harm [NNH] 22; 95% CI, 12–200). The most common adverse events were dry mouth, dizziness, and blurred vision. No serious adverse events were reported in either treatment arm in any of the trials.

**Discussion:** Antispasmodics have anticholinergic or calcium-channel blocking properties, which may help in IBS by relaxing smooth muscles in the gut (134). Although the quality of evidence is very low, antispasmodics have been used in IBS in primary care for a long time and there is some evidence for efficacy in improving IBS symptoms. However, there are a wide variety of medications, and not all were effective. Of the four antispasmodics available in Canada, hyoscine, pinaverium, and dicyclomine (dicylcoverine), were shown to be effective, while trimebutine was not. Therefore, the consensus group suggested the use of only these three antispasmodics. In light of the fact that the evidence was very weak, the potential for anticholinergic side effects, and the higher quality evidence for other alternative treatments, the consensus group suggested that these agents generally be reserved for use in patients who fail other treatments or whose symptoms are best served by an agent taken as needed for abdominal pain.

**Statement 21: We recommend offering IBS patients low-dose tricyclic antidepressants to improve IBS symptoms.**

GRADE: Strong recommendation, high-quality evidence. Vote: strongly agree, 83%; agree, 17%

**Statement 22: We suggest offering IBS patients SSRIs to improve IBS symptoms.**

GRADE: Conditional recommendation, moderate-quality evidence. Vote: strongly agree, 25%; agree, 75%

**Key evidence:** For this consensus, a prior systematic review and meta-analysis (121), was updated with one new RCT (135) for a total of 18 RCTs (n=1127) (125). The majority of trials had small sample sizes, and had an unclear risk of bias. Two-thirds used Rome (I, II, III) criteria for diagnosis of IBS, all but one were double-blind, and seven provided data on the type of IBS patients included.

Overall, antidepressants were significantly more effective than placebo in treating IBS symptoms (RR of IBS symptoms not improving 0.66; 95% CI, 0.57–0.76; *P<*0.00001; NNT 4; 95% CI, 3–6) (125). There was a trend toward heterogeneity between studies, but this was not statistically significant.

Subanalyses according to type of antidepressant class demonstrated significant effects on IBS symptoms with both tricyclic antidepressants (TCAs) (12 studies; RR of IBS not improving 0.65; 95% CI, 0.55–0.77; *P<*0.00001; NNT 4; 95% CI, 3–6) and selective serotonin reuptake inhibitors (SSRIs) (7 studies, RR 0.68; 95% CI, 0.51–0.91; *P=*0.01; NNT 4; 95% CI, 2.5–20) compared with placebo.

Overall adverse event data were available from seven RCTs. The pooled incidence of adverse events was significantly greater among patients who received antidepressants versus those who received placebo (31.3% versus 16.5%; RR 1.63; 95% CI, 1.18–2.25; NNH 9; 95% CI, 5–111). No serious adverse events were reported in any of the trials. Drowsiness and dry mouth were more common in patients who received TCAs than those who received placebo.

**Discussion:** There was good-quality evidence demonstrating that antidepressants overall, TCAs, and SSRIs are effective in improving IBS symptoms and abdominal pain. Possible mechanisms of action of antidepressants in gastrointestinal disorders may include effects on gut transit times, and central and peripheral pain sites, as well as antiinflammatory and analgesic properties (136, 137). TCAs have been extensively studied and have demonstrated efficacy in multiple chronic pain conditions (e.g., neuropathic pain, headaches, low back pain, fibromyalgia), while there is much less support for SSRIs for the treatment of pain (136).

In the clinical trials the TCAs studied included amitriptyline, desipramine, doxepin, imipramine, and trimipramine, and the SSRIs included citalopram, fluoxetine, paroxetine (121, 135). However, the choice of antidepressant should consider a number of disease and medication factors. TCAs have been shown to prolong gut transit times and constipation is a common adverse event, whereas SSRIs may decrease transit time (138). Therefore, TCAs may be more effective in IBS-D (139), and SSRIs in IBS-C (140). However, most of the RCTs did not analyze results according to IBS type. In addition, because TCAs have demonstrated benefits for chronic pain (136), they may be useful in patients with IBS-C once constipation is controlled with other treatments. Because antidepressants in IBS may be used as a short-term treatment trial, the choice of SSRIs should consider that discontinuation syndrome can be more frequent or severe with paroxetine (141, 142).

The effect of antidepressant treatment on coexistent depression or anxiety in IBS patients is controversial. No significant correlations between depression scores and improvements in IBS symptoms have been demonstrated (121). In IBS trials, the doses of SSRIs were similar to those used to treat depression, whereas the doses of TCAs were considerably lower than therapeutic antidepressant doses (121). It has been suggested that the benefits of SSRIs in IBS may be related to improvements in overall well-being (143), but improvements in IBS have been independent of change in mood (121, 143).

From the patient perspective, patients may interpret the prescription of an antidepressant as a dismissal of their symptoms as psychological, or those experiencing mood symptoms, may expect the antidepressant to improve these symptoms (143). Therefore, patient education on the nature of IBS, and the potential effects of antidepressants is crucial for adherence.

The consensus group suggested that SSRIs may be preferred over TCAs for patients with IBS-C and comorbid depression, because of the adverse event profile of TCAs and the fact that SSRIs are used at therapeutic antidepressant doses; however, there is little specific evidence for this. For gastroenterologists who do not feel comfortable prescribing antidepressants, they may want to consider referring patients back to their primary care providers for treatment.

**Statement 23: We suggest AGAINST offering diarrhea-predominant IBS patients continuous loperamide use to improve IBS symptoms.**

GRADE: Conditional recommendation, very low-quality evidence. Vote: strongly agree, 17%; agree, 83%

**Figure 4.**
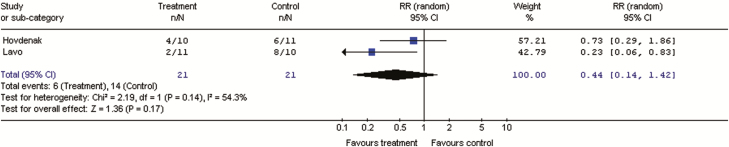
Forest plot of RR of IBS not improving in RCTs of loperamide versus placebo in IBS-D or IBS-M

**Key evidence:** Data were available from two double-blind RCTs including 42 patients with clinically defined IBS-M or IBS-D and compared loperamide with placebo for three to 13 weeks (144, 145). Pooled results showed no significant improvement with loperamide over placebo for global IBS symptoms (RR of IBS not improving 0.44; 95% CI, 0.14–1.42; *P=*0.17) (Figure 4). Loperamide was associated with a significant effect on stool frequency (RR of stool frequency not improving was 0.2 (95% CI, 0.05–0.90; p=0.01) and stool consistency (100% of patients improved versus 20–45%, *P=*0.006) compared with placebo. Beneficial effects on stool frequency, stool consistency, and urgency were also demonstrated in two other RCTs that were not included in the GRADE analysis because of lack of adequate global symptom results (146, 147).

Overall incidence of adverse events with loperamide was similar to placebo in the two included trials (144, 145).

**Discussion:** Loperamide is an opioid receptor agonist that has been shown to decrease peristalsis and fluid secretion, resulting in delayed intestinal transit and decreased loss of fluids and electrolytes (146, 148). Loperamide significantly improved diarrheal symptoms, but has not been shown to consistently improve global IBS symptoms or abdominal pain (144–147). In addition, common side effects of loperamide can include abdominal pain, bloating, nausea, vomiting, and constipation) (149).

Patients will sometimes use loperamide prophylactically when social situations or travel make it inconvenient to have diarrhea, or when participating in a stressful situation that is known to exacerbate diarrhea. Although there is no data on intermittent use, the consensus group acknowledged that some patients may find this strategy useful, and therefore, suggested against continuous loperamide use only.

**Statement 24: We suggest AGAINST offering diarrhea-predominant IBS patients cholestyramine to improve IBS symptoms.**

GRADE: Conditional recommendation, very low-quality evidence. Vote: strongly agree, 8%; agree, 83%; neutral, 8%

**Key evidence:** There is little evidence for the use of cholestyramine in unselected patients with IBS-D. A systematic review, of data on the use of cholestyramine in IBS-D patients also included patients with chronic diarrhea, and only reported response rates in patients with evidence of bile acid malabsorption (BAM) as identified by 7-day SeHCAT scanning (tauroselcholic [^75^selenium] acid) retention rates (150). Pooled data from 15 studies showed a dose-response to cholestyramine according to severity of malabsorption: severe BAM 96%, (<5% retention), moderate BAM 80% at (<10% retention) and mild BAM 70% (<15% retention) (*P=*0.007 for trend). Data were not reported for patients with a negative SeHCAT, who were generally not given a therapeutic trial of cholestyramine.

A systematic review conducted for this consensus identified 15 case-series’ studies in patients with IBS-D (n =1223), but did not identify any RCTs. None of the case-series reported dichotomous data on the proportion of patients responding to cholestyramine with a clear definition of response in unselected IBS-D patients.

**Discussion:** While there was some evidence of symptomatic response in a select group of patients with IBS-D or chronic diarrhea who had idiopathic BAM, efficacy rates decreased with decreasing severity of BAM. The data were very low quality, and may not be generalizable to IBS patients without BAM. Therefore, based on the lack of data in unselected patients with IBS-D, the consensus group suggested against this treatment.

*No recommendation C: The consensus group does not make a recommendation (neither for nor against) offering diarrhea-predominant IBS patients one course of rifaximin therapy to improve IBS symptoms.*

**Key evidence:** For this consensus, a prior systematic review of antibiotics in IBS (7) was updated with two additional RCTs (151, 152) for a total of seven RCTs (n=2654) (119). Patients had non-constipated IBS (predominantly IBS-D).

Overall, rifaximin was significantly more effective than placebo in treating IBS (RR of IBS symptoms not improving 0.82; 95% CI, 0.72–0.95, *P=*0.006; NNT 9; 95% CI, 6–12.5); however, there was significant heterogeneity between trials. The effect remained significant in an analysis that included only the four RCTs with low risk of bias (n=1996; RR 0.87; 95% CI, 0.82–0.93; NNT 11; 95% CI, 8–20) with no significant heterogeneity (153–155).

The incidence of overall adverse events was not significantly greater among patients in the rifaximin group compared with the placebo group (three studies; RR 0.70; 95% CI, 0.42–1.16).

**Discussion:** Rifaximin is a non-systemic broad-spectrum antibiotic derived from rifamycin, which targets the gut (156, 157). Rifaximin has been associated with a low risk of development of bacterial resistance, or cross-resistance with rifampin (157). The mechanisms of action of rifaximin in IBS have not been clearly identified, but may alter the microbiome thus reducing gas production (157, 158).

There was moderate-quality evidence of a beneficial effect of rifaximin over placebo, which continued to be apparent at the end of follow up (three to six months). In addition, one trial suggested efficacy with a repeat course in IBS patients who had relapsed after previously responding to rifaximin (152).

There were a number of concerns around recommending the widespread use of an antibiotic for the management of IBS. Rifamycins are important in the treatment of serious infections, and the potential for antibiotic resistance and cross-resistance with rifaximin is a serious issue. There were also concerns around the poorly defined mechanism of action in IBS, the cost, and the potential for overuse. In addition, although rifaximin has been FDA approved for the treatment of IBS-D in US (156), it has not been approved by Health Canada for this use. As a result there is little experience in Canada with this agent for IBS. From a patient’s perspective, the use of an antibiotic can be confusing, because it may lead patients to believe that they have an infection that will be ‘cured’ after the course of therapy.

Because of these concerns, the consensus group was unable to recommend for or against rifaximin treatment at this time, despite data demonstrating efficacy in IBS. To minimize over use, the consensus group suggested that if this strategy is to be tried, patient should be referred to a gastroenterologist for treatment.

**Statement 25: We suggest offering diarrhea-predominant IBS patients eluxadoline to improve IBS symptoms.**

GRADE: Conditional recommendation, moderate-quality evidence. Vote: strongly agree, 8%; agree, 67%; neutral, 25%

**Figure 5.**
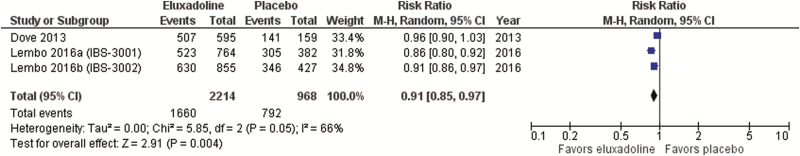
Fores*t* plot of RR of IBS not improving in RCTs of eluxadoline versus placebo in IBS-D

**Key evidence:** The efficacy of eluxadoline in IBS has been reported in three RCTs (n=3235) (159, 160). All three of the included trials used Rome III criteria to define IBS-D, and all were at low risk of bias. In the pooled analysis, eluxadoline was significantly more effective than placebo (RR of IBS not improving 0.91; 95% CI, 0.85–0.97; *P*=0.004; NNT 12.5; 95% CI, 8–33) (Figure 5); however, there was significant heterogeneity between trial results. Eluxadoline had no statistically significant effect on abdominal pain (RR of no improvement 0.95; 95% CI, 0.89–1.02; p=0.06) although there was a trend to benefit, but did improve stool consistency (RR of no improvement 0.88; 95% CI, 0.80–0.96; p<0.01; NNT 10; 95% CI, 6–25).

Overall adverse event data were provided in all three RCTs. The pooled incidence of patients reporting at least one adverse event in the two RCTs reported by Lembo et al was 59% with eluxadoline and 56% with placebo (160). In the other RCT, the rates of adverse events were 48% with eluxadoline and 49% with placebo (159). The most common adverse events with eluxadoline were constipation, nausea, and vomiting (159, 160). Serious adverse reactions of pancreatitis and sphincter of Oddi spasm have been reported (<1% of patients).

**Discussion:** Eluxadoline is a synthetic opioid receptor modulator, with high-affinity for the μ-opioid receptors, and the δ-opioid receptors in the GI tract only. In contrast, loperamide is only active at the μ-opioid receptor (161). The drug was approved by Health Canada in January 2017 for the treatment of IBS-D (162), but only became available in late April, therefore, at the time of this consensus there was little or no clinical experience with this agent in Canada.

There was moderate-quality evidence that eluxadoline had a beneficial effect over placebo, for overall IBS symptoms. Effects were modest with response rates at 26 weeks of about 30% with eluxadoline compared with 20% with placebo (160). The treatment effect of eluxadoline over placebo was observed within the first week and was maintained throughout the 26-week trials (160).

There are a number of safety concerns with eluxadoline, and the drug is contraindicated in patients with biliary duct obstruction, cholecystectomy, alcoholism, pancreatitis, hepatic impairment, and chronic or severe constipation (162). In the clinical trials the majority of serious adverse events (pancreatitis and sphincter of Oddi spasm) occurred in patients with pre-existing conditions including absence of a gallbladder or excessive alcohol consumption (160).

However, among patients with IBS this is an important contraindication because these patients are at 2- to-3-times higher risk of cholecystectomy (4.6% to 12.4%) compared with the general population (2.4% to 4.1%) (163, 164).

Although there was moderate-quality evidence for efficacy, the potential safety issues and the lack of clinical experience with the drug led the consensus group to make only a conditional suggestion in favour of this treatment at this time. In addition, they suggested that if this treatment is to be used, the patient should be referred to a gastroenterologist, and the treatment trial should be of limited duration (e.g., three months).

**Statement 26: We suggest AGAINST offering constipation-predominant IBS patients osmotic laxatives to improve OVERALL IBS symptoms.**

GRADE: Conditional recommendation, very low-quality evidence. Vote: agree, 92%; neutral, 8%

**Key evidence:** Two RCTs that fulfilled inclusion criteria for this consensus, compared polyethylene glycol 3350 (PEG) and placebo in a total of 181 patients with Rome (II or III) diagnosed IBS-C (165, 166). Compared with placebo, there was a statistically significant increase in the number of bowel movements in one study (166), but not in the other (165), and there was no significant improvement in abdominal pain in either study. PEG was generally well tolerated with the most common adverse event being abdominal pain (165, 166).

**Discussion:** PEG is a large polymer that acts as an osmotic laxative. In the study meeting inclusion criteria for this consensus there was no evidence of benefit of PEG in IBS-C patients (165). Another larger RCT (n=139) using a PEG plus electrolytes formulation, found an improvement in the number of bowel movements but no effect on abdominal discomfort/pain. In addition, there is evidence that PEG is effective in chronic idiopathic constipation (CIC) (7).

While there is little evidence that osmotic laxatives (e.g., PEG) will improve overall IBS symptoms, some evidence suggests it has beneficial effects on constipation. Therefore, the consensus group suggested against the use of osmotic laxatives for overall symptoms. However, as was the case with loperamide for diarrhea (see statement 23), the consensus group acknowledged that these agents may be useful in some IBS-C patients, particularly as adjunctive therapy in patients who have improved on other treatments but continue to have constipation.

**Statement 27: We suggest AGAINST offering constipation-predominant IBS patients prucalopride to improve OVERALL IBS symptoms.**

GRADE: Conditional recommendation, very low-quality evidence. Vote: strongly agree, 33%; agree, 58%; neutral, 8%

**Key evidence:** A systematic review including eight RCTs, provided high-quality evidence that prucalopride, a 5-HT_4_ agonist, was effective in patients with CIC (71.1% of patients failed to respond to prucalopride versus 87.4% with placebo; NNT 5; 95% CI, 4–8) (7). However, it has not been evaluated in RCTs in IBS-C patients. A less selective 5-HT_4_ agonist, tegaserod, which has been withdrawn from the market for safety reasons, demonstrated efficacy in IBS-C in a meta-analysis of 11 RCTs (167). There was evidence of publication bias or other small study effects.

**Discussion:** While prucalopride has demonstrated improvements in constipation symptoms in patients with CIC, there is currently no evidence that it will improve overall symptoms in IBS-C patients. In addition, adverse events were more common with prucalopride compared with placebo, including headache, nausea, and diarrhea (7).

Based on the lack of evidence of benefits in IBS patients, the consensus group suggested against this treatment for overall IBS symptoms, but this does not preclude the targeted use of this agent for constipation symptoms.

**Statement 28: We suggest offering constipation-predominant IBS patients lubiprostone to improve IBS symptoms.**

GRADE: Conditional recommendation, moderate-quality evidence. Vote: agree, 83%; neutral, 17%

**Figure 6.**
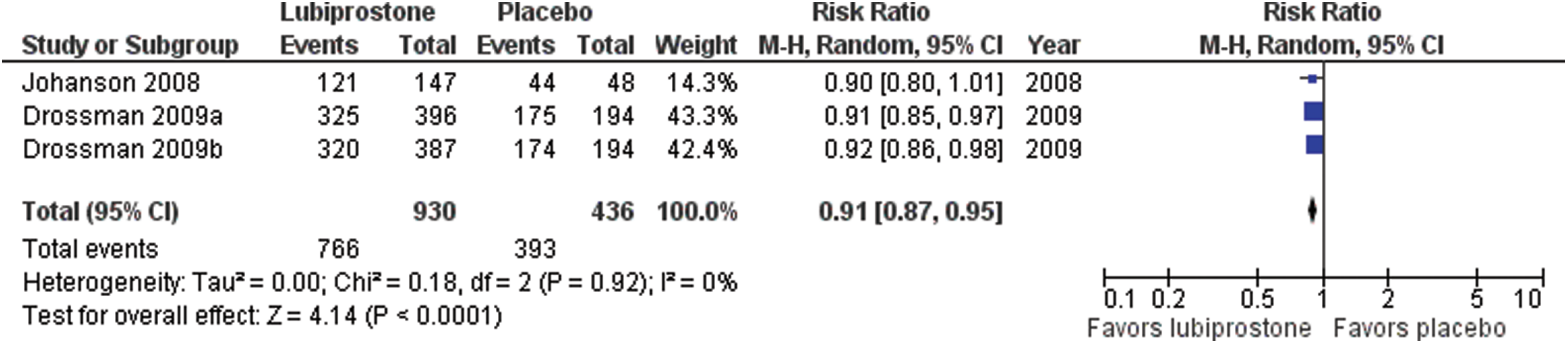
Forest plot of RR of IBS not improving in RCTs of lubiprostone versus placebo in IBS-C

**Key evidence:** Evidence for lubiprostone is available from three RCTs in IBS-C patients (*n=*1366) (168, 169). All three of the trials used Rome II criteria to define IBS-C, and all were at low risk of bias. One trial was a dose-ranging, phase IIb study that assessed lubiprostone 8–24 µg bid, and the other two were phase III studies using a dose of eight µg bid. In the pooled analysis, lubiprostone was significantly more effective than placebo (RR of IBS not improving 0.91; 95% CI, 0.87–0.95; *P<*0.0001; NNT 12.5; 95% CI, 8–25). (Figure 6),

Data on overall adverse events were provided in all three of the trials (168, 169), but were pooled only for the two phase III studies (169). In the phase IIb study and the pooled phase III studies, there were no significant differences in the incidences of overall adverse events between lubiprostone and placebo (168, 169). The phase IIb study reported significantly higher rates of gastrointestinal adverse events (e.g., diarrhea and nausea) particularly at higher doses (168), but this was not seen in the pooled phase III studies using the eight µg bid only (169).

**Discussion:** Although there was moderate-quality evidence that lubiprostone significantly improved overall IBS symptoms, the effect on abdominal pain was significant for the first two months, but not the third month of treatment in the phase IIb study (168). Lubiprostone activates a specific chloride channel (ClC-2) in the GI tract, and thus enhances intestinal fluid secretion and increases GI transit, which may mediate its beneficial effects on constipation symptoms (170). In light of the fact that this treatment is expensive (171), and there are no comparative studies to evaluate whether it will be more effective than other less expensive treatments, the consensus group made a conditional suggestion in favour of using lubiprostone in IBS-C patients.

**Statement 29: We recommend offering constipation- predominant IBS patients linaclotide to improve IBS symptoms.**

GRADE: Strong recommendation, high-quality evidence. Vote: strongly agree, 83%; agree, 17%

**Figure 7.**
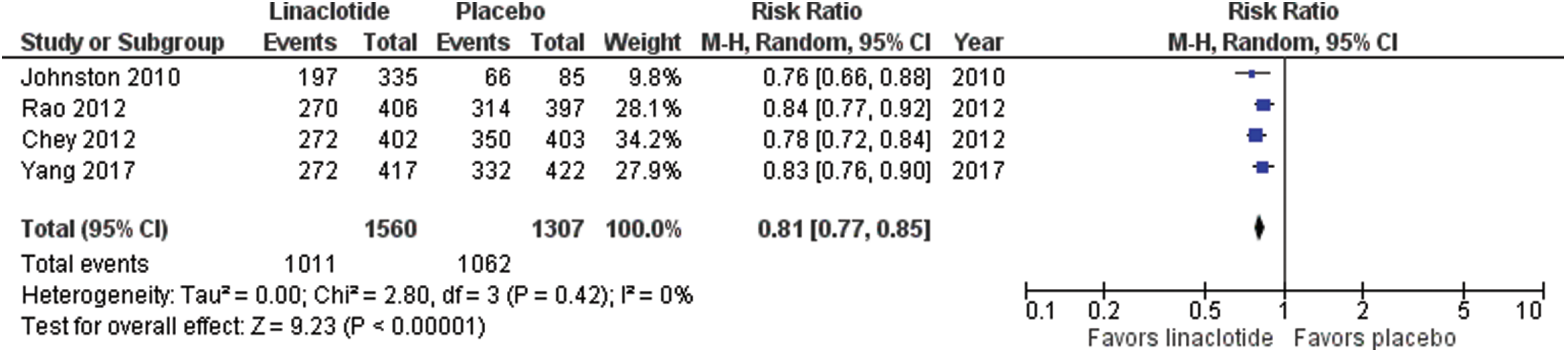
Forest plot of RR of IBS not improving in RCTs of linaclotide versus placebo in IBS-C

**Key evidence:** For this consensus, a prior systematic review and meta-analysis (7), was updated with one additional RCT (172), for a total of four RCTs (n=2867). All trials used Rome criteria (II or III) to define IBS-C, and all were at low risk of bias. In the pooled analysis, linaclotide was significantly more effective than placebo (RR of IBS not improving 0.81; 95% CI, 0.77–0.85; *P<*0.00001; NNT 6; 95% CI, 5–8) (Figure 7). Linaclotide also significantly improved abdominal pain compared with placebo (RR of abdominal not improving 0.82; 95% CI, 0.75–0.89; p<0.001; NNT 8; 95% CI, 5–14).

Overall adverse event data were available from three RCTs. The pooled incidence of any adverse event was significantly greater among patients who received linaclotide versus those who received placebo (49.6% versus 45.2%; RR 1.10; 95% CI, 1.01–1.19; NNH 12). Linaclotide was associated with higher rates of diarrhea (4 studies; RR 6.81; 95% CI, 4.69–9.90; NNH 7; 95% CI, 6–11), and flatulence (2 studies; RR 2.27; 95% CI, 1.18–4.36; NNH 50; 95% CI, 23–167) compared with placebo.

**Discussion:** There was high-quality evidence that linaclotide was effective in improving overall IBS symptoms for IBS-C patients. Linaclotide also significantly improved abdominal pain, abdominal bloating, and bowel symptoms (172–175). Linaclotide is a minimally-absorbed, synthetic peptide that acts on the guanylate cyclase C receptors locally in the gut. It stimulates fluid secretion, increases colonic transit, and modulates afferent pain sensors (171, 176). Although there was a relatively high incidence of diarrhea (10–20%), it infrequently resulted in treatment discontinuation. Over 4000 patients were treated with linaclotide in RCTs, including over 2000 patients treated for one year or longer; most adverse events were mild to moderate in intensity, and the incidence of serious adverse events was low (177).

Although, linaclotide is a relatively expensive treatment (171), the high-quality evidence for improvements in multiple IBS symptoms, and the likely low risk of long-term serious adverse events, led the consensus group to make a strong recommendation in favour of using linaclotide in IBS-C patients.

## FUTURE RESEARCH

The heterogeneous nature of IBS and the lack of specific treatments makes the management challenging. Further research is needed on identifying treatments that will manage both specific and global IBS symptoms. Previous studies have evaluated symptom subgroups according to Rome criteria but studies should evaluate objective parameters such as inflammatory markers, microbiome or metabolomics profiles that might better identify IBS patients that will respond to a specific therapy.

Because IBS symptoms may be related in part to alterations in the microbiota, more research is needed to assess the benefits of altering the microbiome for therapeutic benefit. The antibiotic rifaximin, probiotics, and certain dietary modifications have shown some efficacy in relieving IBS symptoms, which may be related to changes in the colonic microbiota. Further research is needed on the pathogenic changes and strategies to target these changes.

While CBT demonstrated significant beneficial effects on IBS symptoms, self-administered CBT, or CBT delivered via the internet did not. Strategies are need to improve delivery and access to therapist administered CBT. In addition, research is needed to find ways to increase the effectiveness of self-administered or online CBT, which will improve access to less costly, less resource intensive therapy.

## SUMMARY

**Figure 8.**
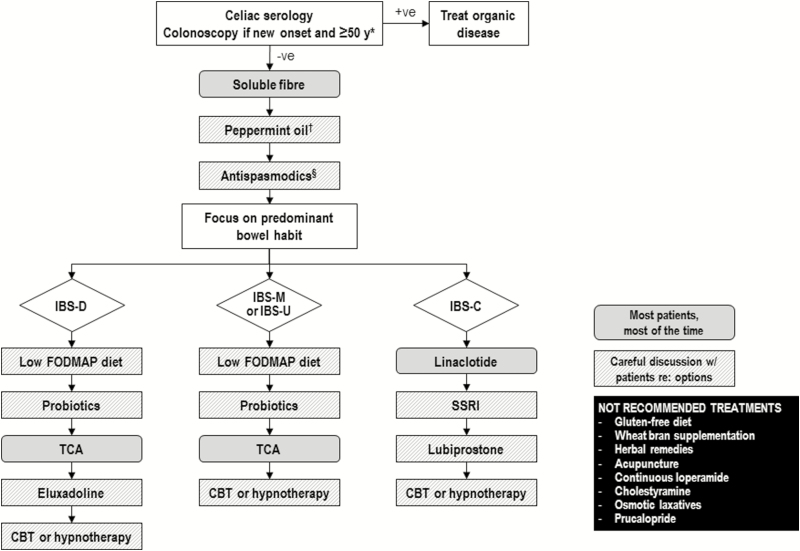
Consensus guided algorithm for the management of IBS. *Reduce age threshold to ≥45 years if female with IBS-D; ^†^if bloating/pain main feature; ^§^if pain main feature.

These guidelines present recommendations for the diagnosis and management of patients with IBS. Consensus was reached on 28 of 31 statements pertaining to the diagnosis and management of IBS (Table 1). An algorithm summarizing the consensus-guided approach to management of patients with IBS is shown in Figure 8.

IBS is diagnosed based on symptoms, with limited use of diagnostic tests; however, serological testing is suggested to exclude celiac disease.

Initial treatment may include a trial of psyllium, but not wheat bran, supplementation to help reduce symptoms. Patients with any IBS whose main symptoms include pain or bloating may benefit from the use of peppermint oil or antispasmodics. A short-term trial of a low FODMAP diet, probiotics, or TCAs, may be useful for patients with IBS-D or IBS-M/U. In addition, patients with IBS-D may benefit from eluxadoline therapy. Pharmacological therapies that may help relieve symptoms for patients with IBS-C include linaclotide, SSRIs, and lubiprostone. Patients with any IBS may also benefit from CBT or hypnotherapy.

These guidelines should help to optimize the use and proper positioning of existing medical therapies and thus improve outcomes in patients with IBS. However, the heterogeneous nature of IBS and the lack of specific treatments continues to make the management challenging. Additional research is needed to identify better treatments for IBS symptoms, and the conclusions of this consensus may subject to change as further data become available and practice patterns evolve.

## Supplementary Material

Supplementary Appendix
