# Supplementary material for: Canadian Association of Gastroenterology Clinical Practice Guideline for the Management of Irritable Bowel Syndrome (IBS)
Source: J Can Assoc Gastroenterol. 2019 Jan 17;2(1):6–29. doi: 10.1093/jcag/gwy071 (PMC6507291; doi:10.1093/jcag/gwy071)
Supplement: Supplementary Appendix [file gwy071_suppl_supplementary_appendix-1.docx]

1. **IBS and Eluxadoline, Searched on Deb 21 2016. N=157**

Database: Embase <1974 to 2016 December 23>, OVID Medline Epub Ahead of Print, In-Process & Other Non-Indexed Citations, Ovid MEDLINE(R) Daily and Ovid MEDLINE(R) 1946 to Present, EBM Reviews - Cochrane Central Register of Controlled Trials <November 2016>, EBM Reviews - Cochrane Database of Systematic Reviews <2005 to December 21, 2016>

Search Strategy:

--------------------------------------------------------------------------------

1 exp eluxadoline/ (115)

2 (eluxadoline or Viberzi or JNJ-27018966).tw,kw. (137)

3 1 or 2 (171)

4 exp Irritable colon/ (28164)

5 exp Irritable Bowel Syndrome/ (28164)

6 (Irritable Bowel Syndrome or IBS).tw,kw. (33821)

7 4 or 5 or 6 (40594)

8 3 and 7 (157)

***************************

1. **IBS and calprotectin. Searched on Jan 25 2017. (update search from 2013 August), n=202**

Database: Embase <1974 to 2017 January 26>, OVID Medline Epub Ahead of Print, In-Process & Other Non-Indexed Citations, Ovid MEDLINE(R) Daily and Ovid MEDLINE(R) 1946 to Present, EBM Reviews - Cochrane Central Register of Controlled Trials <November 2016>, EBM Reviews - Cochrane Database of Systematic Reviews <2005 to January 25, 2017>

Search Strategy:

--------------------------------------------------------------------------------

1 (Irritable Bowel Syndrome or IBS).tw,kw. (33011)

2 calprotectin.tw,kw. (5715)

3 1 and 2 (475)

4 limit 3 to ed=20130801-20170130 use ppez [Limit not valid in Embase,CCTR,CDSR; records were retained] (51)

5 limit 3 to dd=20130801-20170130 use oemezd [Limit not valid in Ovid MEDLINE(R),Ovid MEDLINE(R) Daily Update,Ovid MEDLINE(R) In-Process,Ovid MEDLINE(R) Publisher,CCTR,CDSR; records were retained] (158)

6 limit 3 to yr="2014 -Current" use coch (1)

7 limit 3 to yr="2014 -Current" use cctr (6)

8 4 or 5 or 6 or 7 (216)

9 remove duplicates from 8 (202)

***************************

1. **IBS and colestyramine. Searched on Feb 08 2017, n=208**

Database: Embase <1974 to 2017 February 10>, OVID Medline Epub Ahead of Print, In-Process & Other Non-Indexed Citations, Ovid MEDLINE(R) Daily and Ovid MEDLINE(R) 1946 to Present, EBM Reviews - Cochrane Central Register of Controlled Trials <January 2017>, EBM Reviews - Cochrane Database of Systematic reviews <2005 to February 08, 2017>

Search Strategy:

--------------------------------------------------------------------------------

1 exp Irritable bowel syndrome/ (27898)

2 exp Irritable colon/ (27898)

3 (Irritable bowel syndrome or IBS or (Rome adj2 Criteria)).tw,kw. (35909)

4 1 or 2 or 3 (42646)

5 (colestyramine or cholestyramine or Questran or Cholybar or Olestyr).mp. (14547)

6 4 and 5 (233)

7 remove duplicates from 6 (208)

***************************

1. **IBS and rifaximin. Searched on March 1 2017. (n=112)**

Database: Embase <1974 to 2017 March 06>, OVID Medline Epub Ahead of Print, In-Process & Other Non-Indexed Citations, Ovid MEDLINE(R) Daily and Ovid MEDLINE(R) 1946 to Present, EBM Reviews - Cochrane Central Register of Controlled Trials <February 2017>, EBM Reviews - Cochrane Database of Systematic Reviews <2005 to March 1, 2017>

Search Strategy:

--------------------------------------------------------------------------------

1 exp Irritable bowel syndrome/ (27898)

2 exp Irritable colon/ (27898)

3 (Irritable bowel syndrome or IBS).tw,kw. (33407)

4 1 or 2 or 3 (40190)

5 rifaximin or Xifaxan.tw. (2753)

6 4 and 5 (502)

7 random$.mp. (3072869)

8 6 and 7 (188)

9 ((exp animals/ or exp animal/ or exp nonhuman/ or exp animal experiment/ or animal model/ or animal tissue/ or non human/) not (humans/ or human/)) or ((rats or mice or mouse or cats or dogs or animal* or cell lines) not (human* or men or women)).ti. (10755590)

10 ((child/ or Pediatrics/ or Adolescent/ or Infant/ or adolescence/ or newborn/) not (adult/ or aged/)) or ((baby or babies or child or children or pediatric* or paediatric* or peadiatric* or infant* or infancy or neonat* or newborn* or new born* or kid or kids or adolescen* or preschool or pre-school or toddler*) not (aged or adult* or elder* or senior or men or women)).ti. (4287974)

11 8 not (9 or 10) (183)

12 remove duplicates from 11 (112)

***************************

**5. IBS and Colonscopy and organic diseases. Searched on March 15 2017. N= 441**

Database: Embase <1974 to 2017 March 21>, OVID Medline Epub Ahead of Print, In-Process & Other Non-Indexed Citations, Ovid MEDLINE(R) Daily and Ovid MEDLINE(R) 1946 to Present, EBM Reviews - Cochrane Central Register of Controlled Trials <February 2017>, EBM Reviews - Cochrane Database of Systematic Reviews <2005 to March 15, 2017>

Search Strategy:

--------------------------------------------------------------------------------

1 exp Irritable bowel syndrome/ (28022)

2 exp Irritable bolon/ (28022)

3 (Irritable bowel syndrome or IBS).tw,kw. (33594)

4 or/1-3 (40401)

5 Colonoscopy/ or sigmoidscopy/ (96778)

6 (colonoscop* or sigmoidscop*).tw,kw. (73310)

7 5 or 6 (115928)

8 4 and 7 (1850)

9 colorectal neoplasms/ or colonic neoplasms/ or rectal neoplasms/ (188189)

10 colon cancer/ or colon tumor/ or large intestine cancer/ or colon adenocarcinoma/ or colon carcinogenesis/ or colon carcinoma/ or colorectal cancer/ or colorectal carcinoma/ or sigmoid carcinoma/ (377512)

11 rectum cancer/ or rectum tumor/ or colorectal carcinoma/ or rectum carcinoma/ (185890)

12 ((colon* or colorectal or rectal or rectum) adj3 (cancer* or carcinoma* or malignan* or tumor* or turmour* or neoplas* or adenocarcinoma*)).tw,kw. (441124)

13 exp Colonic Polyps/ or exp colon polyp/ or exp colorectal polyp/ or exp rectum polyp/ (29571)

14 ((colon* or colorectal or rectal or rectum) adj3 (adenoma* or polyp*)).tw,kw. (42832)

15 organic.tw,kw. (493009)

16 or/9-15 (1064608)

17 8 and 16 (529)

18 remove duplicates from 17 (441)

***************************

1. **IBD and diet (Gluten free or low FODMAP). Searched on April 12 2017. N= 1998**

Database: Embase <1974 to 2017 April 13>, OVID Medline Epub Ahead of Print, In-Process & Other Non-Indexed Citations, Ovid MEDLINE(R) Daily and Ovid MEDLINE(R) 1946 to Present, EBM Reviews - Cochrane Central Register of Controlled Trials <March 2017>, EBM Reviews - Cochrane Database of Systematic Reviews <2005 to April 12, 2017>

Search Strategy:

--------------------------------------------------------------------------------

1 exp Irritable bowel syndrome/ (28258)

2 exp Irritable colon/ (28258)

3 (Irritable bowel syndrome or irritable colon* or IBS).tw,kw. (34832)

4 1 or 2 or 3 (41261)

5 exp diet, gluten-free/ (8860)

6 exp gluten free diet/ (8860)

7 ((gluten* adj2 free) or glutens).tw,kw. (11497)

8 exp fructose oligosaccharide/ or exp polyol/ or exp fructose/ or exp galactose oligosaccharide/ (242440)

9 exp diet/ (590580)

10 (FODMAP or FODMAPs or saccharides or oligosaccharide or disaccharide or monosaccharide).tw,kw. (63021)

11 exp diet restriction/ (145695)

12 exp fructan/ (8749)

13 (polyol or polyols or diet restriction or dructo-oligosaccharides or galacto-oligosaccharides or fructans or fructose or galactans or lactose or sorbitol or mannitol or xylitol or maltitol).tw,kw. (166833)

14 exp carbohydrate diet/ or exp Dietary Carbohydrates/ (46682)

15 exp sweetening agent/ (298613)

16 sweetener*.tw,kw. (7427)

17 (diet or diets or dietary or nutrition or food).tw,kw. (1788646)

18 or/5-17 (2589439)

19 4 and 18 (6544)

20 randomized controlled trial.pt. (879623)

21 controlled clinical trial.pt. (183129)

22 random:.mp. (3123681)

23 placebo:.mp. (802774)

24 trial.ab. (1204795)

25 groups.ab. (4274341)

26 double-blind*.mp. or blind*.tw. (936966)

27 clinical trial:.mp. (2709808)

28 or/20-27 (8427422)

29 19 and 28 (2844)

30 remove duplicates from 29 (1998)

1. **IBS and lactose Breath test or lactose intolerance. Searched on May 10, 2017**

Database: Embase <1974 to 2017 May 12>, OVID Medline Epub Ahead of Print, In-Process & Other Non-Indexed Citations, Ovid MEDLINE(R) Daily and Ovid MEDLINE(R) 1946 to Present, EBM Reviews - Cochrane Central Register of Controlled Trials <April 2017>, EBM Reviews - Cochrane Database of Systematic Reviews <2005 to May 10, 2017>

Search Strategy:

--------------------------------------------------------------------------------

1 exp Breath Tests/ (32109)

2 (breath test* or (lactose adj2 breath)).tw,kw. (18737)

3 1 or 2 (37949)

4 exp Irritable bowel syndrome/ (27051)

5 (Irritable bowel syndrome or IBS).tw,kw. (33707)

6 4 or 5 (40419)

7 3 and 6 (1196)

8 (Alactasia or hypolactasia or ((lactose or dairy product or milk sugar) adj3 (intolerance or malabsorption))).tw,kw. (4818)

9 exp lactose intolerance/ (6869)

10 8 or 9 (8246)

11 7 and 10 (240)

12 (lactose adj2 breath).tw,kw. (508)

13 6 and 12 (92)

14 11 or 13 (249)

15 remove duplicates from 14 (175)

***************************

1. **IBS and glucose breath test or SIBO. Searched on May 10 2017. N=506.**

Database: Embase <1974 to 2017 May 12>, EBM Reviews - Cochrane Central Register of Controlled Trials <April 2017>, EBM Reviews - Cochrane Database of Systematic Reviews <2005 to May 10, 2017>, Ovid MEDLINE(R) Epub Ahead of Print, In-Process & Other Non-Indexed Citations, Ovid MEDLINE(R) Daily, Ovid MEDLINE and Versions(R)

Search Strategy:

--------------------------------------------------------------------------------

1 exp Breath Tests/ (32109)

2 (breath test* or (glucose adj2 breath)).tw,kw. (18673)

3 1 or 2 (37947)

4 (small intestinal bacterial or SIBO).tw,kw. (2091)

5 3 and 4 (1272)

6 conference abstract.pt. or Congresses as Topic/ (2634994)

7 5 not 6 (852)

8 remove duplicates from 7 (506)

***************************
